# Predicting occupant head displacements in evasive maneuvers; tuning and comparison of a rotational based and a translational based neck muscle controller

**DOI:** 10.3389/fbioe.2023.1313543

**Published:** 2024-01-12

**Authors:** Emma Larsson, Johan Iraeus, Bengt Pipkorn, Jonas Östh, Patrick A. Forbes, Johan Davidsson

**Affiliations:** ^1^ Mechanics and Maritime Sciences, Chalmers University of Technology, Göteborg, Sweden; ^2^ Autoliv Research, Vårgårda, Sweden; ^3^ Volvo Cars Safety Centre, Gothenburg, Sweden; ^4^ Department of Neuroscience, Erasmus MC, University Medical Center Rotterdam, Rotterdam, Netherlands

**Keywords:** active human body model, pre-crash, omni-directional control, SAFER HBM, controller tuning

## Abstract

**Objective:** Real-life car crashes are often preceded by an evasive maneuver, which can alter the occupant posture and muscle state. To simulate the occupant response in such maneuvers, human body models (HBMs) with active muscles have been developed. The aim of this study was to implement an omni-directional rotational head-neck muscle controller in the SAFER HBM and compare the bio-fidelity of the HBM with a rotational controller to the HBM with a translational controller, in simulations of evasive maneuvers.

**Methods:** The rotational controller was developed using an axis-angle representation of head rotations, with x, y, and z components in the axis. Muscle load sharing was based on rotational direction in the simulation and muscle activity recorded in three volunteer experiments in these directions. The gains of the rotational and translational controller were tuned to minimize differences between translational and rotational head displacements of the HBM and volunteers in braking and lane change maneuvers using multi-objective optimizations. Bio-fidelity of the model with tuned controllers was evaluated objectively using CORrelation and Analysis (CORA).

**Results:** The results indicated comparable performance for both controllers after tuning, with somewhat higher bio-fidelity for rotational kinematics with the translational controller. After tuning, good or excellent bio-fidelity was indicated for both controllers in the loading direction (forward in braking, and lateral in lane change), with CORA scores of 0.86−0.99 and 0.93−0.98 for the rotational and translational controllers, respectively. For rotational displacements, and translational displacements in the other directions, bio-fidelity ranged from poor to excellent, with slightly higher average CORA scores for the HBM with the translational controller in both braking and lane changing. Time-averaged muscle activity was within one standard deviation of time-averaged muscle activity from volunteers.

**Conclusion:** Overall, the results show that when tuned, both the translational and rotational controllers can be used to predict the occupant response to an evasive maneuver, allowing for the inclusion of evasive maneuvers prior to a crash in evaluation of vehicle safety. The rotational controller shows potential in controlling omni-directional head displacements, but the translational controller outperformed the rotational controller. Thus, for now, the recommendation is to use the translational controller with tuned gains.

## 1 Introduction

Vehicle safety has improved over the past decades (Borsos et al., 2012; Forman et al., 2019) due to the introduction of safety systems like seat belts and airbags (Kahane, 2015). Furthermore, vehicles undergo rigorous evaluation through consumer safety tests (Kullgren et al., 2019). Although high ratings of occupant safety in consumer tests correlate with lower risk of injury in real-life crashes (Kullgren et al., 2019; Teoh and Arbelaez, 2022), these tests currently represent only a fraction of the potential real-world crash scenarios. For instance, the seated postures and positions of the anthropometric test devices in consumer tests are predefined, and include a limited number of postures and positions [e.g., (Euro NCAP, 2021)]. In real-life, however, initial occupant postures and positions vary (Reed et al., 2020), and vehicle crashes are often preceded by an evasive maneuver (Scanlon et al., 2015; Riexinger and Gabler, 2018; Sander and Lubbe, 2018; Riexinger et al., 2019), leading to greater variability in occupant position (Ólafsdóttir et al., 2013; Kirschbichler et al., 2014; Ghaffari et al., 2018; Reed et al., 2019). To account for this, human body models (HBMs) that include active musculature have been developed. These active HMBs can predict occupant responses to evasive maneuvers (Meijer et al., 2012; Kato et al., 2018; Devane et al., 2019; Larsson et al., 2019; Martynenko et al., 2019).

For all impact directions, one of the most important injuries to predict are injuries sustained to the head (Pipkorn et al., 2020). Specifically, brain injury risk is known to be correlated with the measured head rotation (Gabler et al., 2016). Furthermore, other studies have shown that the head rotation during a crash is influenced by the initial conditions, like spine curvature (Poulard et al., 2014; Poulard et al., 2015; Sato et al., 2017) and initial head yaw orientation (Leledakis et al., 2021). These findings indicate that to accurately predict the risk of head injuries during a crash, active HBMs used to simulate pre-crash maneuvers must predict both head orientation and position.

Despite its importance for injury prediction, HBM predictions of head rotation have either not been reported or failed to reproduce experimental responses with volunteers when simulating evasive maneuvers or low-speed impacts. For example, in the validation of the active THUMS (v6), in this paper referred to as THUMS, in low-speed frontal impacts, only head translations were reported (Kato et al., 2018). Similarly, only head translations were reported in the validation of A-THUMS-D (Martynenko et al., 2019). In a later study using the same model in a different loading environment (Wochner et al., 2022), similar predictive outcomes were reported for head translations as for head rotations. When using the GHBMC simplified model (M50-OS+Active), in this paper referred to as the GHBMC, to simulate low-speed frontal impacts, head translational acceleration was captured with better bio-fidelity compared to head rotational accelerations (Devane et al., 2019). Furthermore, when comparing the results for the same model with active control gains tuned to volunteer tests, only head translations were reported (Devane et al., 2022). Using the MADYMO Active Human Model, in this paper referred to as the MADYMO model, to simulate low-speed frontal impact, head translations could be predicted well, while rotational accelerations were underpredicted (Valdano et al., 2021). In another study with the MADYMO model, head rotations were predicted well in slalom maneuvers (Mirakhorlo et al., 2022). The SAFER HBM has previously been rated with fair to good bio-fidelity in both braking and lane changing, however, head roll and yaw were overpredicted in lane change (Larsson et al., 2019).

In addition to head posture and position, capturing muscle activity can be important for accurate simulation of evasive maneuvers because neck muscle forces can influence predicted neck injuries. For instance (Deng et al., 2021), found that for frontal impacts, increasing neck muscle activity increased neck injury criteria (Nij) values in high-velocity impacts (>60 km/h), while for lower velocity impacts (<60 km/h) neck injury criteria (Nij) values were decreased. Further, in (Östh et al., 2022), a decrease in neck injury criteria (Nij) together with a slight increase in lumbar spine forces was seen when including muscle activity to simulations of frontal impacts. Since neck muscle forces can be influential in injury prediction, including human-like activity, with both human-like load sharing and activation level, is essential. Because several muscles have the same line of action, and humans co-contract surrounding muscles to stabilize joints (Marieb and Hoehn, 2019), human intermuscular load sharing cannot be determined by only considering the muscle geometry (Vasavada et al., 2002; Fice et al., 2018). In some studies, the muscle geometries have been combined with a minimization function that minimizes energy consumption of muscle activation to determine a muscle activation pattern (de Bruijn et al., 2016).

Humans have been suggested to utilize two reflexes for head posture maintenance (Keshner, 2009), the vestibulocollic reflex (VCR) (Binder et al., 2009d; Keshner, 2009), which sense head linear and rotational accelerations and aims to maintain the head posture in space, and the muscle stretch reflex (cervicocollic reflex (CCR) for cervical muscles) (Binder et al., 2009c; Keshner, 2009), which aim to maintain the length of the muscles. When using active HBMs to simulate evasive maneuvers, there are different strategies available for controlling the neck muscle activations, most of them emulating the functionality of VCR. In both GHBMC and THUMS, the neck muscles are activated based on head rotation relative to torso rotation (Kato et al., 2018; Devane et al., 2019). In the MADYMO model, neck muscle activation is also determined by head rotation, with the reference posture determined by the user (Meijer et al., 2012). In the A-THUMS-D model (Martynenko et al., 2019), muscles are activated using both closed loop feedback and open loop based on muscle length, and head position and orientation is indirectly controlled using the lengths of individual muscles. Although the GHBMC, THUMS and MADYMO model all respond to head rotations, the x, y, and z rotations are calculated separately and specified muscle activity from the three rotations are superimposed. In A-THUMS-D, intermuscular load sharing (distribution of muscle activation between muscles in the system, e.g., neck muscles) is not controlled. Importantly, none of these models include neck muscle controllers that respond to head rotations, with intermuscular load sharing based on data from humans. In the SAFER HBM v10, head rotations are indirectly controlled for by activating neck muscles based on head translations relative to T1, with muscle activity levels based on activity recorded in human subject experiments (Larsson et al., 2019; Ólafsdóttir et al., 2019; Östh et al., 2022).

In SAFER HBM (Pipkorn et al., 2021), active muscle controllers have been implemented in the neck and lumbar regions (Östh et al., 2022), as well as for the upper and lower extremities (Östh et al., 2015). For neck and lumbar musculature, the model’s muscle activation is based on spatial tuning patterns of human volunteers during omnidirectional, horizontal plane, whole-body seat translations (Ólafsdóttir et al., 2015). Using SAFER HBM (v9) (Larsson et al., 2019), model kinematics were compared to kinematics from volunteers in braking, lane changing and lane changing with braking maneuvers. Four different controllers were compared, three head translational controllers with different reference coordinate systems (to emulate VCR or muscle stretch reflexes/CCR depending on which coordinate system was used), and one muscle length feedback controller (to emulate muscle stretch reflexes/CCR). It was concluded that the best predictions of kinematics were obtained with the controller that was closest to emulate VCR, compared to controllers that were closer to muscle spindle reflexes/CCR. While the results indicated good overall kinematic predictive capabilities, the controller gains in the model were not tuned after modelling and controller updates, leaving room for further improvement. In addition, the model was not capable of predicting head rotations, especially head yaw rotations in lane change, which were overpredicted. This may be because the implemented controllers only respond to translations and not to head rotations. Therefore, the first objective of this study was to implement a controller in the SAFER HBM that responds to head rotations, emulating VCR, with intermuscular load sharing based on recorded muscle activity from volunteers. The second objective of this study was to tune the gains of both the rotational and translational controllers to compare the predictive capabilities of the tuned controllers. The overall aim of this study was to increase the bio-fidelity of the SAFER HBM response to simulated evasive maneuvers.

## 2 Materials and methods

**FIGURE 1:**
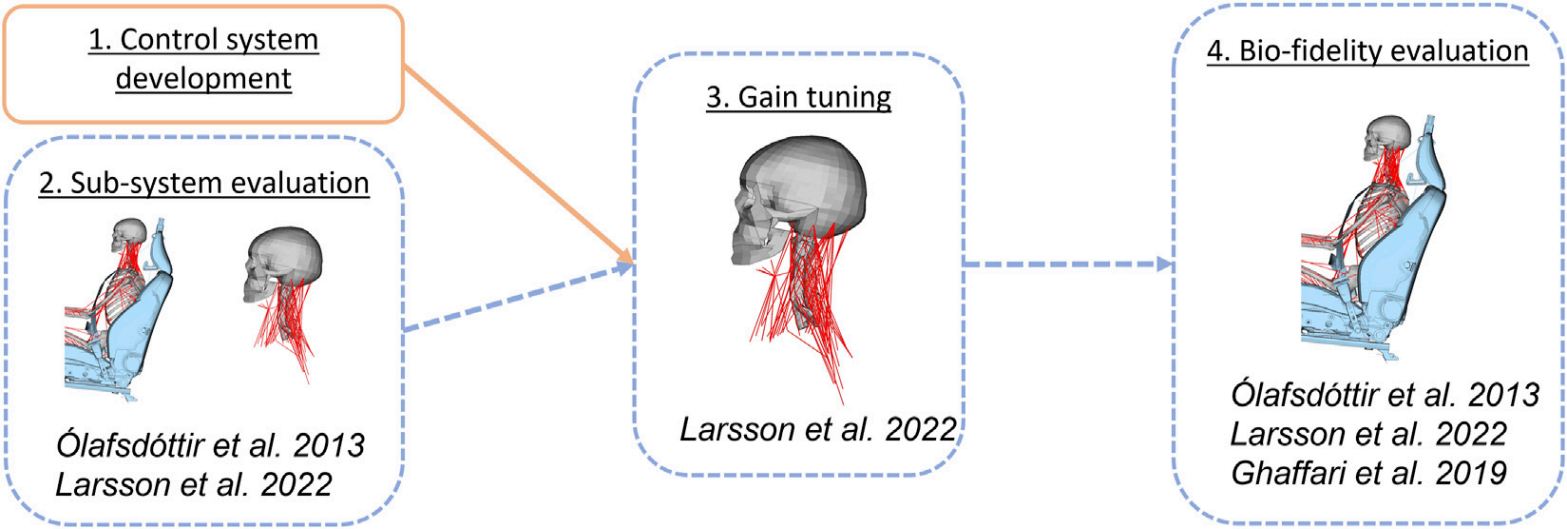
Method overview presenting the four steps included. Volunteer data used in each part of the study in italic, see section for each step for details. Activities in orange were related to the first objective and dashed blue to the second objective.

This study was conducted in four steps, see Figure 1. Step one addressed the first objective, and steps two to four addressed the second objective. First, a control system responding to head rotations was developed. Second, an evaluation of head kinematics from a sub-system with an isolated head-neck was conducted. Simulations of the sub-system were compared to simulations using a full-body model, to determine if it was possible to reduce computational cost in the subsequent tuning by using a sub-system in tuning. Third, both the rotational and translational controllers were tuned using the isolated head-neck sub-system. Finally, the bio-fidelity of the model with tuned controllers was evaluated by comparing head displacements and neck muscle activity in full-body model simulations to volunteers subjected to braking and lane change.

All simulations were performed with LS-DYNA MPP R12.0.0 Double Precision (SVN version 148978, LST, Livermore, CA, USA). Pre and post processing was done in ANSA and MetaPost v18 (BETA CAE Systems, Switzerland), MATLAB R2020b (The Mathworks Inc., Natick, MA, US), and LS-PrePost V4.9 (LST, Livermore, CA, USA).

### 2.1 Maneuvers (used in steps 2, 3 and 4)

Occupant kinematics, muscle activity and vehicle accelerations from braking and lane change experiments, collected in two sets of volunteer tests were used when tuning and evaluating the controllers. These were two braking maneuvers, one from (Ólafsdóttir et al., 2013) and one from (Larsson et al., 2022), and one lane change maneuver (Ghaffari et al., 2019; Larsson et al., 2022), originally presented in (Ghaffari et al., 2018). All kinematics were reported in a vehicle fixed coordinate system with the positive x-axis in the vehicle forward direction, and z-axis in the vertical downward direction.

In the (Ólafsdóttir et al., 2013) study, 20 volunteers (9 female, 11 male) were exposed to braking maneuvers (peak acceleration 11 m/s^2^, duration 2.5 s), while seated in the passenger seat of a Volvo V60. Head and torso sagittal plane kinematics, belt force and position and muscle activity (eight muscles, measured bilaterally) were reported, for males and females separately. The muscle activity was reported as time histories and as average activity for a specified time interval (1.6–1.8 s). In the current study, kinematics and average muscle activity from male participants were used for comparison.

In the (Ghaffari et al., 2018; Ghaffari et al., 2019; Larsson et al., 2022) study, 21 volunteers (12 female, 9 male) were exposed to vehicle maneuvers such as braking (peak acceleration 9 m/s^2^, duration 2 s) and lane changes (a right turn—referred to as first phase of maneuver—followed by a left turn -referred to as second phase of maneuver -, peak acceleration level 6 m/s^2^, duration 2 s), while seated in the passenger seat of a Volvo V60. Head and torso translational and rotational kinematics as well as belt forces and positions were parameterized using principal component analysis (PCA), and reported as predictive models using belt, sex, stature, age and BMI as covariates (Larsson et al., 2022). Muscle activity during the lane change was reported as time histories together with average activities over three pre-defined phases (baseline phase, right turn (first phase) and left turn (second phase); corresponding to approximately <0s, 0–1s and 1–2s respectively) (Ghaffari et al., 2019). In this simulation study, the predictive models were used to create kinematic responses for a male with a stature of 175 cm, age of 45 years and BMI of 25 kg/m^2^, restrained by a standard inertia reel belt. Average muscle activity from male participants was used for comparison.

### 2.2 Simulation setup (used in steps 2, 3 and 4)

In this study, an isolated head-neck sub-system model was extracted from the SAFER HBM (step 2) and used for gain tuning (step 3), while full HBMs were used to evaluate the bio-fidelity after gain tuning (step 4). SAFER HBM v10, or a sub-system of this model, was used for all simulations (Pipkorn et al., 2021).

#### 2.2.1 Sub-system simulations (used in steps 2 and 3)

The sub-system consisted of the isolated head and neck, from T1 vertebra level and up. The soft tissue interface nodes were constrained to T1. Muscles in the neck controller that attached to parts not included in the sub-system (e.g., clavicle) were kept in the model, with inferior origin or insertion nodes constrained to T1. Translational and rotational boundary conditions were applied to T1 (Putra et al., 2021). For the tuning simulations (step 3), the displacements were derived from volunteer experiments and for the sub-system evaluation simulations (step 2) the displacements were extracted from the full-body simulations.

The tuning of the sub-system was performed in two phases; a pre-simulation phase with gravity loading only, with T1 stationary, where controllers were initialized over a period of 250 ms, and a loading phase. For the braking simulations, the loading phase was 2,250 ms long, and for lane change simulations, the loading phase was 1,450 ms.

#### 2.2.2 Full body simulations (used in steps 2 and 4)

Braking and lane change simulation setup was the same as used in previous simulations of the same experiments (Larsson et al., 2019). In brief, the simulation environment consisted of a deformable seat, a rigid footrest, and a 2-D seat belt model, including buckle and D-Ring. The retractor was modelled with a retractor element, with prescribed loading and unloading curves. Accelerations were prescribed on a single element at vehicle center of gravity. The seat model has previously been validated using low-load quasi-static indentation tests, by comparing predictions from the model to results from tests with a physical seat from the same vehicle model (Östh et al., 2012). The same belt attachment points and belt properties as in (Larsson et al., 2019) were used.

The simulations consisted of two phases run seamless; a pre-simulation phase with gravity loading only, where controllers were initialized (750 ms for braking and 650 ms for lane change), followed by a loading phase where evasive maneuvers were imposed. For the braking simulations, the loading phase was 2300 ms long (i.e., 3,050 ms total simulation time), and for lane change simulations, the loading phase was 2,150 ms long (i.e., 2,800 ms total simulation time).

### 2.3 Control system development (step 1)

**FIGURE 2:**
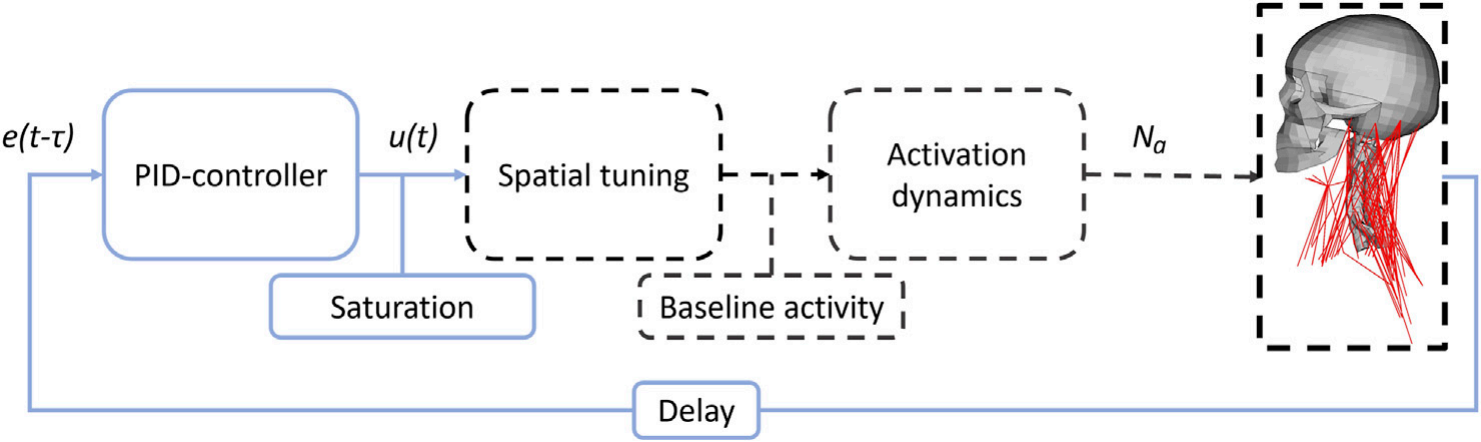
Controller flowchart. e(t*-τ*) shows the delayed error signal, u(t) the PID response and N_a_ the muscle activation signals. Blue solid lines indicate signals and actions common for all muscles, while black dashed lines indicate muscle group specific signals and actions.

Both the existing translational controller from SAFER HBM (Larsson et al., 2019) and the new rotational controller presented here, follow the same structure adapted from (Ólafsdóttir et al., 2019). The controllers emulate reflexes that aim to maintain the head orientation in space (Manzoni, 2009) such as the sensory feedback provided by the vestibular system, Figure 2. A deviation, or error, from a reference position was measured in the model. This error, with a delay *τ*, e(t*-τ*) to represent neural transitioning and processing, was fed through a Proportional-Integral-Derivative (PID) controller, which produces a response u(t), as an activation level common for all muscles. The response was saturated if the PID controller produced a response above the maximum activity of unity. After saturation, the intermuscular load sharing was determined by scaling the common activity level to muscle specific signals using spatial tuning patterns (STPs). Baseline activity was added to the muscle specific signals to represent muscle activity during quiet sitting, based on minimum activity in spatial tuning patterns. This baseline activity was included in the full duration (gravity settling and maneuver). The last step was the activation dynamics, consisting of two first order differential equations that filtered and delayed the response (Ólafsdóttir et al., 2019), based on work from (Winters and Stark, 1985).

#### 2.3.1 Translational controller

In the SAFER HBM v10, a controller based on translational displacements was implemented (Larsson et al., 2019; Östh et al., 2022). The angular deviation of a vector/link from T1 to head center of gravity (calculated from head translations of head center of gravity relative to T1) between the reference position (before maneuver) and current position was used to activate the muscles. The controller was named angular position feedback (APF) controller, but has been referred to as translational controller (Tra) in this study. This controller aims to maintain the head position on the torso, relative the gravity field. The sensory input to the controller is based on translational displacements of head relative torso, in a coordinate system that rotates with T1 in z-rotations. Humans can sense head translational accelerations and orientation in gravitational field through the otoliths (Marieb and Hoehn, 2019), which are part of the vestibular system. If head rotations and displacements are coupled, this would also imply a strategy aimed at maintaining head orientation in the gravity field. Humans can sense angular accelerations through the semicircular canals, which also are part of the vestibular system (Marieb and Hoehn, 2019).

**FIGURE 3:**
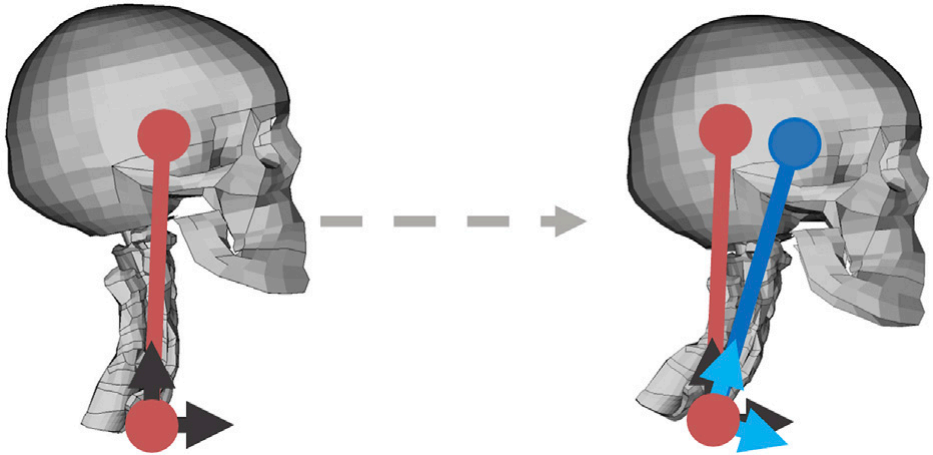
Translational controller, with the reference position in red, and current position in blue. The black arrows show the HBM fixed/global yaw coordinate system with x direction forward and z direction upward, and the light blue arrows show the T1 local coordinate system. Before any movement, the coordinate systems were aligned. Left figure shows reference position, and right figure shows a position during braking.

The deviation from reference, in a coordinate system that follows T1 in rotations around global z axis (yaw), Figure 3, was used as the error signal for the PID controller, Figure 2. Distribution of activation between muscles included in the system (intermuscular load sharing scaling) was done based on displacements in a local coordinate system attached to T1, Figure 3, and direction of displacement in the local x-y plane was used to scale the muscle activity from a general activity level to muscle specific signals, Figure 2, by using experimentally derived STPs (Ólafsdóttir et al., 2015).

#### 2.3.2 Rotational controller

The new rotational control system was implemented based on the concept from the translational controller but driven by changes in head rotation instead of translation. This controller, in this study abbreviated Rot, aims to maintain the head orientation in space. The sensory input to the controller was based on rotational displacements relative a global coordinate system. Humans can sense head rotational accelerations through the semicircular canals (Marieb and Hoehn, 2019), and orientation in gravity field through the otoliths (Marieb and Hoehn, 2019). If head rotations and displacements are coupled, this would also imply a strategy aimed at maintaining head position on the torso.

**FIGURE 4:**
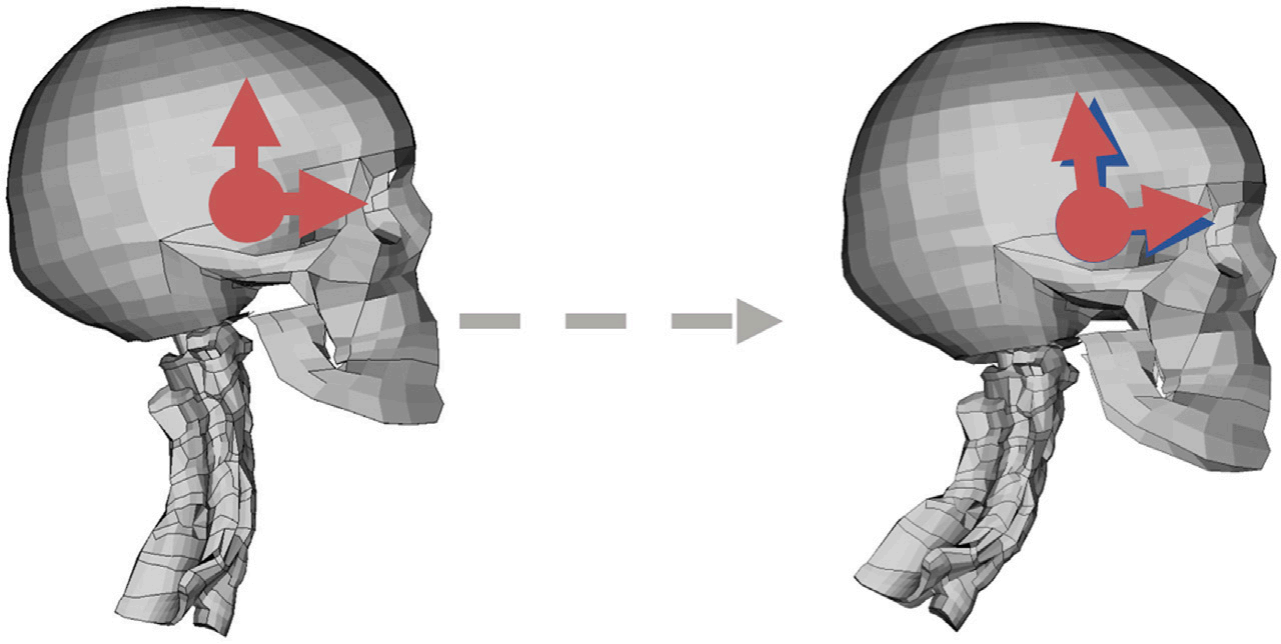
Rotational controller coordinate system depicted in red; the horizontal arrow shows the local x axis, and the vertical arrow shows the local z axis. The blue coordinate system in the right figure shows reference coordinate system, determined at the reference time. The dashed gray arrow goes from the reference to current position.

The rotational displacements of the head CoG were extracted, relative to a globally fixed coordinate system, initially aligned with the head, Figure 4. These rotational displacements were converted to the axis-angle representation (Diebel, 2006), in which the rotation was described by one rotation magnitude and one axis around which the rotation has occurred. The axis-angle representation was calculated from the nodal rotational displacements of the head CoG node, relative to a globally fixed coordinate system. First, a rotation matrix R was calculated from successive rotations around z, y, and x, Supplementary Equation S1. Secondly, the axis ( v ) and angle ( θ ) were calculated from that rotation matrix (Råde and Westergren, 1998), Supplementary Equations S2, S3. The change in angle θ was used as input signal to the PID controller (i.e., error signal e in Figure 2), and the axis v was used in the intermuscular load sharing.

For 11 muscle groups, Supplementary Table S4, recorded muscle activity from three different experiments were combined to form STPs. There is one specific pattern per muscle group, which means there are 11 STPs, Supplementary Table S4, with the structure described in Supplementary Table S2. For each muscle group, the STP consisted of 26 data points, as defined by eight flexion/extension and lateral bending combinations at three different axial rotation levels (right axial rotation, no axial rotation, left axial rotation), and two pure axial rotations. For most of these 26 directions, two or more experimental results were available (all data available is shown in Supplementary Table S6). Data derived from dynamic experiments were preferred over values derived from isometric experiments (Ólafsdóttir et al., 2019) because dynamic and isometric activation patterns can differ (Siegmund et al., 2007), and the intended model use was in dynamic conditions. Furthermore, data normalized using maximum voluntary isometric contractions (MVICs) were preferred over non-MVIC normalized data (Burden, 2010), because MVIC normalization allows for comparison between muscles. The STPs were created for right side muscles, and then mirrored for the left side. For the current application, all STPs describe muscle activity when returning from that orientation.

##### 2.3.2.1 Perturbations (P)

In the experiments published in (Ólafsdóttir et al., 2015), 8 volunteers (6 male, 2 female) were exposed to translation perturbations along the horizontal plane in 8 different directions. Muscle activity was recorded from nine muscles with indwelling wire electrodes (of which eight were used, Supplementary Table S3) and was normalized with recorded MVICs. Since muscle activity was recorded in dynamic conditions and normalized using MVIC, it was used wherever available, Table V.

##### 2.3.2.2 Additional experiments

Two additional experiments were conducted, where two male participants generated dynamic head movements (Experiment 1) and isometric neck muscle contractions (Experiment 2). The protocol was explained prior to the experiment and both subjects gave their written informed consent. The experiment conformed to the Declaration of Helsinki and was approved by the University of British Columbia’s Clinical Research Ethics Board. Muscle activity was recorded bilaterally using indwelling wire electrodes from eight different neck muscles, Supplementary Table S3. Muscle activity was normalized to the peak response within each experiment. For both experiments, data from the left and right-side muscles were combined by mirroring the left-side data to produce a single average across both sides. This mirroring was performed by mirroring both rows and columns in Table V. For additional details regarding subject preparation and electrode placement, see (Forbes et al., 2018), where an additional experiment from the same recording session was presented. The full spatial tuning matrices for both experiments are available in the Supplementary Material.

In Experiment 1, which was dynamic, subjects were seated facing a board with lights arranged in two concentric rings. Subjects began by looking at the center point. When one of the lights in the rings was illuminated, the subjects oriented their head with their view to the light, as fast as possible. When the light turned off, the subjects rapidly returned their head orientation and view to the center of the rings. The muscle activity when returning to center was used in the current study, because the controllers intend to return the head back to initial position. This experiment was used to construct each muscle group’s STP when data from perturbations experiment (Ólafsdóttir et al., 2015) was unavailable, Supplementary Table S2.

In Experiment 2, which was isometric, subjects were seated with their head rigidly fixed to a helmet that was attached to a load cell. The subjects generated isometric torques at approximately 0.15 MVIC during 1 s, in the 26 directions described by the STPs. Subjects were given visual feedback of the forces and torques needed to generate the desired loads for each direction. The same test procedure (except for MVIC collection and number of repetitions) and recording equipment was used in (Fice et al., 2018). Because these experiments were isometric, these data were only used where no other data was available, Supplementary Table S2.

#### 2.3.3 Combining data

Since multiple data sources were used to construct the STPs, a method of combining the different data sources was created. First, the average right-side muscle data (from average across both sides), created as described in the section above, was mirrored to create left-side muscle STPs, in the same way as left-side recorded activity was mirrored to create the average activity across both sides. Secondly, muscle activity for each direction in the STP was normalized with maximum recorded muscle activity in that direction, considering both right and left side muscle activity. Resulting STPs, Supplementary Table S5, were visualized using 5 polar plots, one polar plot for each of the 5 levels of axial rotation (i.e., left and right pure axial rotation, combinations of left and right axial rotation and other rotations, no axial rotation) in Supplementary Table S2. Since different muscles were included in the experiments, a substitute muscle pattern from a muscle with similar function was used whenever needed, Supplementary Table S3. Muscles were grouped according to Supplementary Table S4, adapted from (Ólafsdóttir et al., 2019).

### 2.4 Sub-system evaluation (step 2)

To determine if it was sufficient to perform controller gain optimizations on a sub-system with an isolated head-neck using T1 kinematics as boundary conditions, a comparison between the full-model and isolated system was performed. The full model SAFER HBM, with baseline (i.e., original, untuned) controller implementation (Pipkorn et al., 2021), was exposed to braking (Ólafsdóttir et al., 2013) and lane change (Larsson et al., 2022). T1 translational and rotational displacements were extracted from the full-model simulations and used as boundary conditions on T1 in the sub-system simulations. Resulting head displacements were compared to the full-model head kinematics using the CORrelation and Analysis (CORA) software (CORAplus 4.0.4 (Gehre et al., 2009)), with settings in Supplementary Table S1.

### 2.5 Gain tuning (step 3)

The gains of the controller (K_p_, K_i_, K_d_) were tuned to minimize the difference in head displacements between simulation models and volunteers. Gain tuning was performed using LS-OPT v7 (LST, Livermore, CA, USA) using multi-objective and multi-load case optimizations. The isolated head-neck model was used in gain tuning. Each optimization included two maneuvers (braking and lane change from (Larsson et al., 2022)) and contained two or four objectives. Three optimizations were performed per controller, with objectives shown in Table 1, resulting in a total of 6 optimizations. The LS-OPT CurveMapping function was used as a curve comparison metric to compare full time series of simulation data (HBM head displacements) to reference data (volunteer head displacements). To prevent the controllers from saturating the muscle activation, an optimization constraint of 99% maximum muscle activation was used, with a modification that added the duration of saturation to the response. This modification ensured that the optimizer could differentiate between simulations with sustained muscle saturation from those with only short periods of muscle saturation.

$$u\left(t\right)=K_{p}e\left(t\right)+K_{i}\int_{0}^{t}e\left(\tau \right)d\tau +K_{d}\frac{de\left(t\right)}{dt}$$ (1)

$$u\left(t\right)=K_{p}\left(e\left(t\right)+\frac{1}{T_{i}}\int_{0}^{t}e\left(\tau \right)d\tau +T_{d}\frac{de\left(t\right)}{dt}\right)$$ (2)

**TABLE 1:** Optimization objectives. For all displacements, the objective was to minimize the difference between volunteer and HBM displacement time histories.

Name	Objective	
	Braking	Lane change
Translation (_T)	Head X translation	Head Y translation
Rotation (_R)	Head Y rotation	Head X rotation
Translation and rotation (_TR)	Head X translation	Head Y translation
	Head Y rotation	Head X rotation

The parallel PID controller formulation, Eq. 1, was used for gain tuning because the gains are independent in this formulation, while the standard form, Eq. 2, was used when estimating reasonable starting values and ranges for tuning, (Paz, 2001), because the used tuning rules are formulated for the standard form.

In all gain tuning loops, a metamodel-based optimization, specifically, the sequential response surface method (SRSM) with domain reduction (NIELEN STANDER et al., 2020), was used. With the metamodeling approach, a small number of simulation results (in this case 7 simulations per iteration) were used to calculate a response surface across the full design space. With the SRSM, for each iteration, a reduction of the design space is made (zooming) while the center of the design space is adjusted (panning), both according to results from previous iteration (NIELEN STANDER et al., 2020). This method has been successfully used in gain tuning optimizations previously (Putra et al., 2021). The metamodel was built using a linear polynomial with D-Optimal point selection. The termination criteria were set to a design change tolerance and objective change tolerance of 0.01 (default) and a maximum of 10 iterations was used. Three parameters (K_p_, K_i_, K_d_) were used in the tuning, Table 2. The proportional part will respond more to larger errors but cannot alone reduce the error to zero since as the error reduces to zero, so does the proportional response. The integral part can reduce the error to zero given sufficient time but will respond slower than the proportional part (Åström and Murray, 2021). The derivative part mainly acts to stabilize the system.

**TABLE 2:** Starting, maximum and minimum controller variables used in the tuning of controller gains, including baseline values from SAFER HBM v10.

Model		Tuning case	Tuning method	KP [1/rad]	KI [1/rad ms]	Theoretically tuned KI [1/rad ms]	KD [ms/rad]
Optimization settings	Starting	Braking and lane change (Larsson et al., 2022)	SRSM with domain reduction, metamodeling	0.1	1e-6		100
	Minimum			0	0		0
	Maximum			2	0.1		1,000
SAFER HBM v10 (Larsson et al., 2019)		Braking (Östh et al., 2015)	Single stage iteration, metamodeling with space filling design (Östh et al., 2015)	1.301	0	9e-4	470
THUMS (Kato et al., 2018)		Low-speed frontal impacts (Kato et al., 2017)	Manual tuning Kato et al., 2017)	8.0	0	0.05	300
GHBMC (Devane et al., 2019)		Low-speed frontal impacts (Devane et al., 2019)	Manual tuning (Devane et al., 2019)	3.0	0	0.009	250
GHBMC (Devane et al., 2022)		Low speed frontal impacts at two levels (Devane et al., 2022)	Single stage iteration, space filling design (Devane et al., 2022)	0.78	0	3e-4	483.27

Maximum values of K_p_ and K_d_ were based on values in the baseline model [SAFER HBM v10 (Larsson et al., 2019)], and defined roughly as double the values in the baseline model. Minimum values for all parameters were set to 0 to allow the optimizer to turn each part off. Since no previous models have used an integration term, maximum K_i_ was calculated using Ziegler-Nichols tuning rules (step response method) (Åström and Murray, 2021). First, the relationship between T_i_ and T_d_ was calculated from the tuning rules (T_i_ = 4T_d_). Secondly, T_d_ from the previous active models was calculated based on K_p_ and K_d_ (T_d_ = K_d_/K_p_). Finally, based on T_d_ and K_p_, the theoretically Ziegler-Nichols step response tuned K_i_ (K_i_ = K_p_^2^/4K_d_) was calculated for the models, Table 2, and the maximum across the similar models (THUMS, 0.05) rounded up to the nearest order of magnitude (0.1) was used as a maximum allowed K_i_ in the gain tuning. For all three parameters, starting values were set to the lower end of the range by trial and error, to ensure that the optimizer found at least one feasible design, i.e., with maximum muscle activity below the defined constraint (the simulation which used all three starting values).

### 2.6 Bio-fidelity evaluation (step 4)

To evaluate the bio-fidelity of the active HBMs with tuned controller gains, full body simulations of braking (Ólafsdóttir et al., 2013) and lane change (Ghaffari et al., 2019; Larsson et al., 2022) with the resulting gains were performed. Head displacements were compared to average experimental volunteer head displacements using CORA (settings and rating in, Supplementary Table S1). Combined overall scores for the head displacements were calculated by weighting the translations and rotations with the peak magnitude of the volunteer displacements, as was done in (Larsson et al., 2019), such that displacements with smaller magnitude were given less importance than displacements with larger magnitudes. Translations and rotations were attributed equal value in the calculation of the overall score. Bio-fidelity was determined to be similar if they obtained the same CORA rating, Supplementary Table S1, and different if a different rating was obtained. The average muscle activity predicted by the model was compared to measured muscle activity of volunteers, with the durations defined in each of the experiments, of left and right-side sternocleidomastoid (SCM) and semispinalis cervicis (SCerv, model)/cervical paravertebral muscles (CPVM, experiments). The N_a_ signal on group level, Figure 2, was used in the comparison. To assess the quality of the of the model’s ability to replicate the data, we classified responses that fell within the average ±1 SD as being “similar” and responses that fell outside as being “dissimilar.”

## 3 Results

### 3.1 Sub-system evaluation (step 2)

The sub-system head displacements were comparable to the displacements from the full model and could thus be used in the gain optimization, with CORA scores for head forward translational displacement in braking and head lateral translational displacements in lane change rated as excellent. It was therefore judged that the sub-system could be used for the gain optimization. For details, see Sub-system evaluation in Supplementary Material.

### 3.2 Gain tuning (step 3)

Most of the tuned controllers stayed below maximum allowed activity constraint (0.99) throughout the simulation, Table 3, with the exception for the rotational controller tuned to rotations (both only rotations and combined with translations). For these controllers, the maximum allowed activity constraint was violated briefly during braking acceleration ramp-up and rebound. All optimizations terminated due to reaching the maximum allowed number of iterations (10). All three tuned translational controllers had similar gains as the baseline model. All three PID parameters had non-zero gains, except K_I_ for Tra_R and Rot_TR. For Rot_R, the proportional gain was lower compared to the other tuned controllers, while the integrational gain was higher compared to the other tuned controllers. For Rot_TR, both K_P_ and K_I_ were lower than for the other controllers.

**TABLE 3:** Tuned controller gains, maximum PID response in braking (B) and lane change (LC), and number of iterations for each optimization.

Controller implementation	Optimization objective (abbreviation for combination of implementation and objective)	Max. PID response		Iterations	KP [1/rad]	KI [1/rad ms]	KD [ms/rad]
		B	LC				
Translational controller	Translation (Tra_T)	0.81	0.89	10	1.218	2e-6	460.9
	Rotation (Tra_R)	0.89	0.96	10	1.515	0.0	511.0
	Translation and rotation (Tra_TR)	0.77	0.84	10	1.203	5.356e-5	431.8
Rotational controller	Translation (Rot_T)	0.79	0.59	10	0.511	2.5e-9	205.9
	Rotation (Rot_R)	1.0	0.98	10	0.084	0.001	331.9
	Translation and rotation (Rot_TR)	1.0	0.65	10	0.146	0.0	401.2

The predicted x displacement of translational controllers in braking was of similar magnitude as for the volunteers, Supplementary Figure S4. For the rotational controllers, the predicted magnitude of x displacements in braking was slightly smaller compared to the volunteers. For all models, the predicted y displacement in lane change was smaller than for the volunteers for almost all models, Supplementary Figure S4. The z displacement in braking was similar for all rotational controllers, some of the translational controllers and volunteers. For some of the translational controllers, some oscillation in head kinematics was induced in the braking maneuver. Convergence plots, Supplementary Figure S5, show that the integral gain was rapidly reduced to the lower end of the allowed range, while for proportional and derivative gain the optima were found in roughly the middle of the allowed range. For rotational controllers (Rot_T, Rot_R, Rot_TR) the ranges for proportional gain were slightly decreasing during the two final iterations, while for the translational controllers (Tra_T, Tra_R, Tra_TR) the ranges for proportional gain were slightly increasing. The range of derivative gains for both controllers remained similar for the final two iterations.

### 3.3 Bio-fidelity evaluation

In both braking and lane change, all tuned translational controller models (Tra_T, Tra_R, Tra_TR) had similar bio-fidelity, and were comparable to the baseline (untuned) controller model, while the rotational controller models (Rot_T, Rot_R, Rot_TR) had lower bio-fidelity in lane change, evaluated by overall CORA score. In braking, compared to baseline, bio-fidelity was similar for Rot_T and Rot_R and lower for Rot_TR.

#### 3.3.1 Braking

**FIGURE 5:**
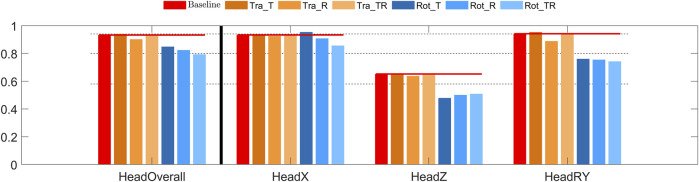
CORA score for head displacement in braking. The leftmost red bars show the CORA scores for the baseline model, added horizontal lines extends these scores to give a reference for the other models. The dashed lines show the thresholds for rating of the CORA scores, for “excellent”, “good”, “fair”, and “poor” CORA scores. From left to right: overall score, head translations (x and z), head rotation (y).

The overall head CORA score indicated good to excellent bio-fidelity for the translational controllers, and fair to good bio-fidelity for the rotational controllers, Figure 5. Head x displacement (the loading direction) was predicted with good bio-fidelity, except for Rot_T showing excellent bio-fidelity. Head z displacement bio-fidelity was fair for the translational controllers and poor for the rotational controllers. Head rotation bio-fidelity was excellent for the controllers with baseline and Tra_T gains, good for the Tra_R and Tra_TR controllers, and fair for the rotational controllers.

**FIGURE 6:**
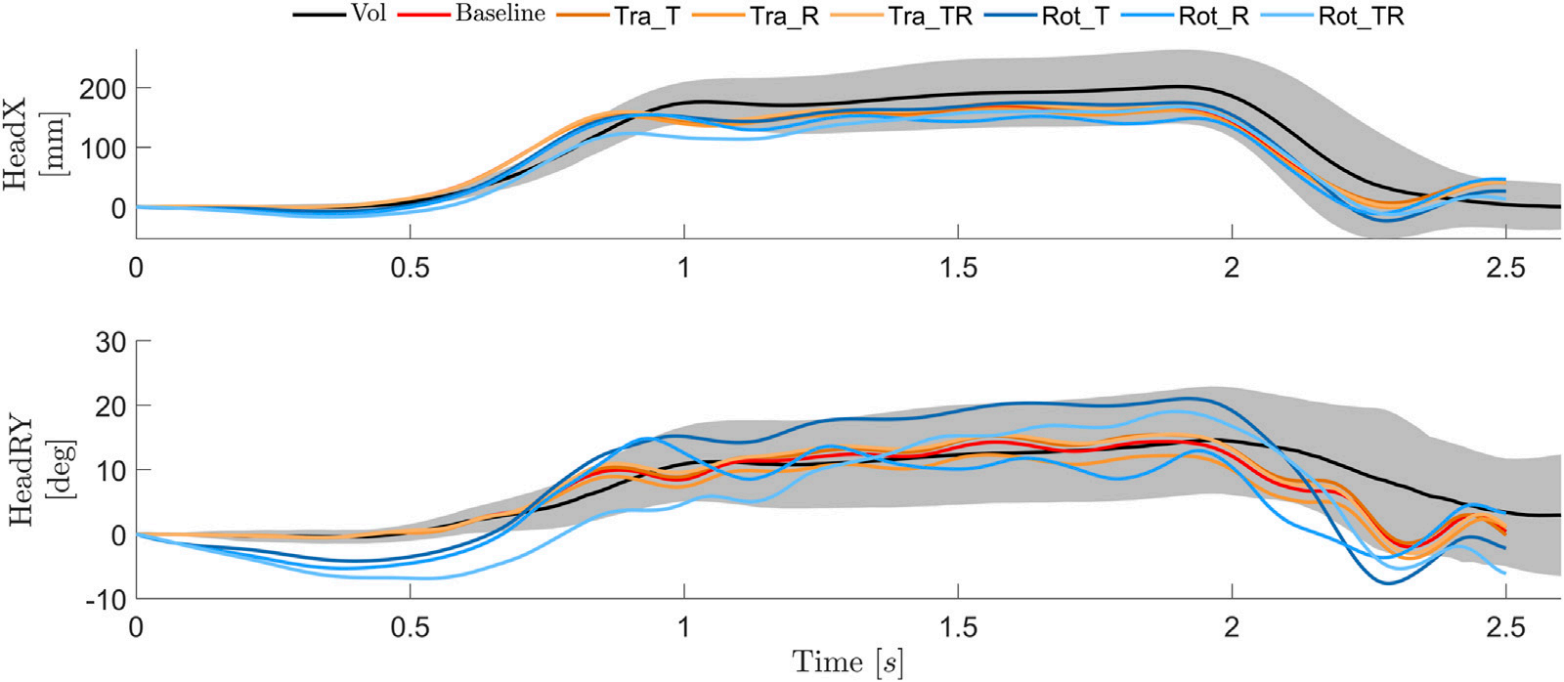
Head translations and rotations for volunteers [average (black) ±1 SD (gray)] (Ólafsdóttir et al., 2013), and simulation models with baseline (red), tuned translational (orange) and tuned rotational (blue) controllers.

During steady state braking (after about 1 s), the predicted head displacement magnitudes were smaller compared to the average volunteer response, but within one SD, for models using both the baseline and tuned controllers, Figure 6. However, the rotational controllers all rotated the head rearward prior to loading onset, and were rearward of the initial position until approximately 0.75 s. The baseline and tuned versions of the translational controller managed to maintain the initial head rotation until the head started to displace (after around 0.5 s). All models exhibit some head y rotation oscillations during 1–2.5 s, which was not observed in human volunteers.

**FIGURE 7:**
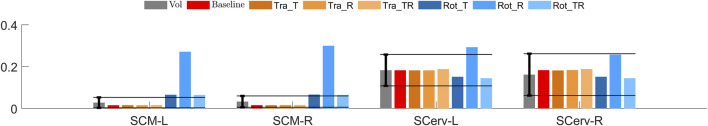
Muscle activity (fraction of MVIC) in braking. Volunteers in gray, with the bar indicating average and the error bars ± 1SD, (Ólafsdóttir et al., 2013), added solid horizontal lines extend the error bars to allow for easier comparison to the models. The red bar shows the activity for the baseline, untuned model, orange bars show tuned translational models and blue shows tuned rotational controller models.

Compared to the volunteers, all simulations using translational controllers produced similar average muscle activity, Figure 7. The Rot T and Rot_TR controller predictions were within ±1 SD for SCerv muscles, while the predicted SCM response was at or slightly outside the +1 SD margin. The Rot_R controller predicted larger muscle activity for SCM, while the predicted SCerv activation was on and outside the +1 SD margin.

#### 3.3.2 Lane change

**FIGURE 8:**
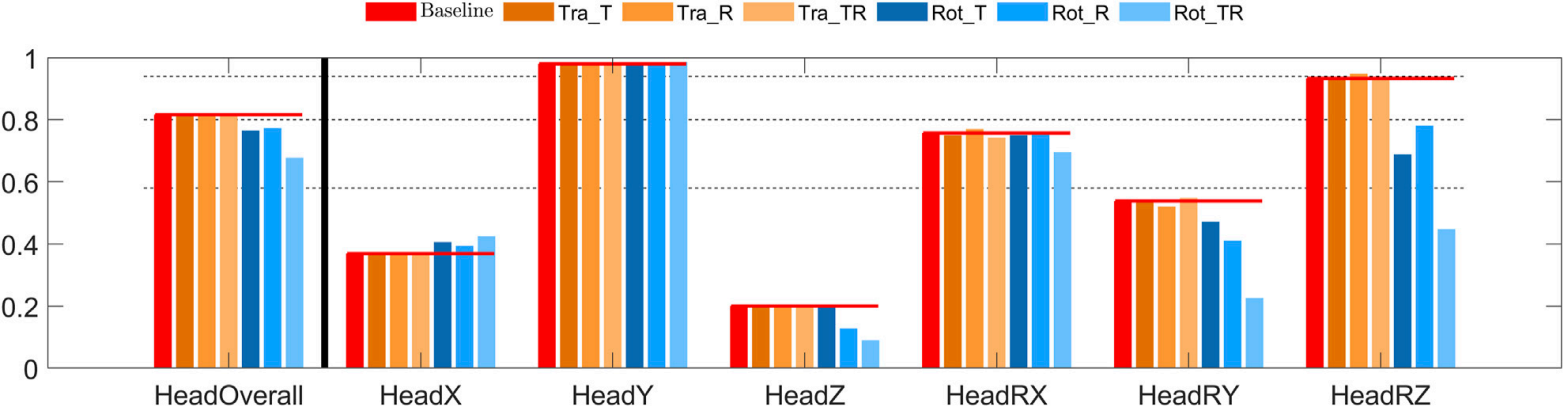
CORA scores for head displacements in lane change. The leftmost red bars show the CORA scores for the baseline model, added horizontal lines extends these scores to give a reference for the other models. The dashed lines show the thresholds for rating of the CORA scores, for “excellent,” “good,” “fair,” and “poor” CORA scores. From left to right: overall score, head translations (x, y, and z), head rotations (x, y, and z).

Compared to the baseline model, the overall head CORA scores were similar for all the tuned translational models, showing good bio-fidelity. The rotational controllers in contrast showed fair bio-fidelity, Figure 8. All models showed poor bio-fidelity in x and z displacements, and excellent bio-fidelity in y displacements (the loading direction). Similarly, x rotations were predicted with fair bio-fidelity and y rotations were predicted with poor bio-fidelity for all models. The z rotation predictions resulted in excellent bio-fidelity for the Tra_R controller, good bio-fidelity for all other translational controllers, fair bio-fidelity for Rot_T and Rot_R controllers, and poor bio-fidelity for the Rot_TR controller.

**FIGURE 9:**
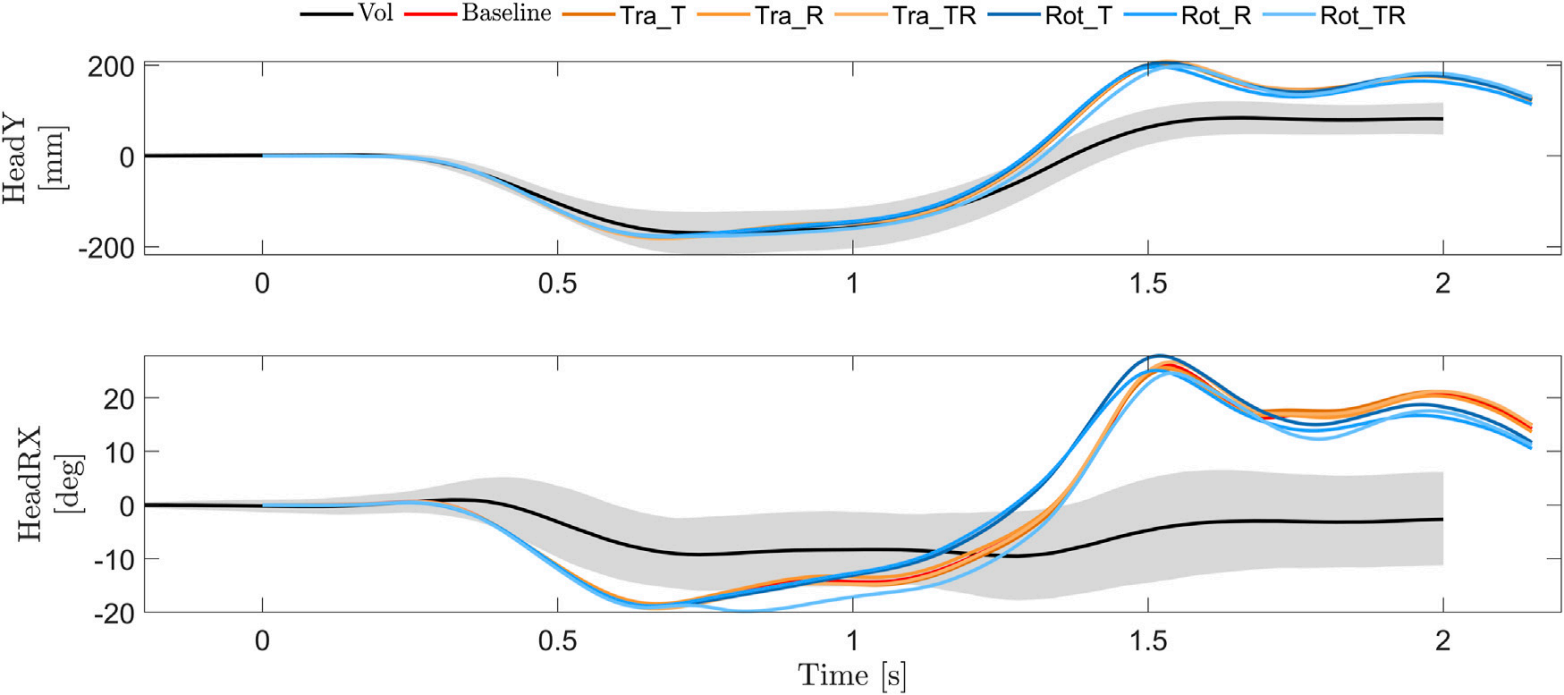
Head and torso lateral (y) translations and head (x) rotations for volunteers (average (black) ±1 SD (gray)) (Larsson et al., 2022), and simulation models with baseline (red) and tuned gains for the translational controller (orange) and rotational controller (blue).

All simulation models predicted similar lateral translations (Head Y), Figure 9. The onset time and shape of the predicted lateral translations were similar to the volunteers in the first phase, however, the peak head lateral translation occurred slightly earlier for all simulation models compared to volunteers, Figure 9. The magnitude of peak lateral translation was similar in the first phase and larger for all models in the second phase. The predicted head x rotation was larger for all models compared to the volunteers, especially in the second phase, where the volunteers on average remained in slightly negative rotation (inboard rotation) while all models predicted head rotations in the positive direction (outboard rotation).

**FIGURE 10:**
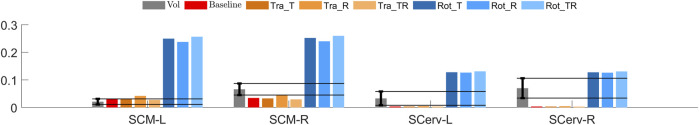
Muscle activity (fraction of MVIC) in lane change. Volunteers in gray, with the bar indicating average and the error bars ± 1SD, (Ghaffari et al., 2019), added solid horizontal lines extend the error bars to allow for easier comparison to the models. The red bar shows the activity for the baseline, untuned model, orange bars the tuned translational models and blue bars the tuned rotational controller models.

Compared to the volunteers, all tuned head rotational controllers predicted higher average muscle activity for SCM and left SCerv during first phase of lane change, Figure 10. All translational models predicted similar activity compared to the volunteers.

## 4 Discussion

In this study, a neck muscle controller that responds to head rotations, with intermuscular load sharing based on data from volunteers, was implemented in the SAFER HBM v10, and compared to the existing translational controller in SAFER HBM. The gains of the rotational and translational controllers were tuned by multi-objective optimization. Three optimizations were performed per controller, aimed at minimizing the difference between simulation and volunteer head kinematics, but using different kinematic components as optimization objectives. Finally, the predictive capabilities of both controllers were evaluated by comparing the model predictions to volunteer data. The results show that all models (translational and rotational controllers, with baseline and tuned gains) predicted the head translation in the loading direction (longitudinal in braking, lateral in lane change) with good to excellent bio-fidelity for both controllers. In braking, using translational controllers, rotational and translational displacements were predicted with equal bio-fidelity, while for rotational controllers in braking and both controllers in lane change, rotational displacements were rated with lower bio-fidelity compared to translational displacements. Overall, slightly better bio-fidelity rating scores were achieved with the translational controllers. Further, even if both controllers had similar performance, the translational controller would have been preferred over the rotational controller because the translational controller is easier to understand, debug and interpret intermediate results from.

In the current implementation, the translational controller responds to horizontal plane translations of the head relative to T1 in a coordinate system that rotates with T1 in yaw while, the rotational controller responds to 3D rotations of the head relative the head initial orientation in a global coordinate system initially aligned with the initial orientation of the head. To allow for simulations of maneuvers where the occupant is rotated during simulation, such as when the vehicle turns, the coordinate system should follow the model in (at least) horizontal plane rotations, as was done for the translational controller, (Larsson et al., 2019). For the rotational controller, implementation of such a coordinate system added extra complexity, and for this study a global coordinate system was used instead. Another alternative could have been to use a completely local coordinate system attached to T1 (Larsson et al., 2019); however, such an approach can lead a controller that falls closer to emulating muscle stretch reflexes rather than the vestibular system reflexes, which was the intention with the new controller. Since the maneuvers simulated in this study contain only small yaw rotations the difference between different coordinate system implementations would have likely been negligible.

Both controllers evaluated in this study used intermuscular load sharing from recorded muscle activity in volunteer tests. This was done because human intermuscular load sharing cannot be determined by only considering the muscle geometry (Vasavada et al., 2002; Fice et al., 2018) as several muscles have the same line of action (Marieb and Hoehn, 2019). However, an alternative approach would be to use optimization to derive activation patterns, where force generation in the model is combined with (metabolic) cost minimization (de Bruijn et al., 2016). This approach could have been beneficial for the rotational controller, where volunteer data was scarce for many of the combined head rotations. Since we had enough volunteer data available to build spatial tuning patterns for the rotational controller, when combining data from different experiments, these data were used instead of deriving patterns based on the model geometry, to ensure that the patterns were based on muscle activity from humans.

The implementation of the translational controller, where head displacements relative to torso displacements were used as input (via the link from T1 to the head center of gravity), is similar to the implementations in GHBMC and THUMS (Kato et al., 2018; Devane et al., 2019), where the head rotations relative to torso rotations are used as input. The rotational controller, where rotations of the head relative to the global coordinate system are used as input, is more similar to one of the implementations (global reference posture) in the MADYMO model (Meijer et al., 2012). None of the implementations in this study are similar to the approach used in the A-THUMS-D (Martynenko et al., 2019), where muscle lengthening is used to activate the muscles. Common for these controllers, including the two controllers used in this study, is that they activate the muscles based on emulations of the human physiology and reflexes, but none of them are detailed implementations of the human reflexes or anticipated goal-oriented movements. A more detailed modelling of VCR and CCR was implemented in (Happee et al., 2017), using an isolated head-neck model. In contrast to the implementations in THUMS, GHBMC, MADYMO, and the controllers in this study, the aim with the detailed implementations was to identify the specific strategies used by volunteers in the reference data, rather than to model the kinematics exhibited by the volunteers subjected to complex vehicle maneuvers. Therefore, that detailed approach was not considered applicable to our study.

The rotational controller tuned using translations showed slightly better bio-fidelity for head forward translational displacement in braking compared to the translational controllers. In contrast, the translational controllers showed better bio-fidelity for rotational kinematics compared to all rotational controllers. This was not expected, since the controller was intended to improve the rotational displacements compared to the translational controller. We also observed that the rotational controllers initiated head rearward rotation (extension) prior to braking initiation, Figure 6. This was likely due to a combination of imbalanced baseline activities and the low proportional gain terms of the controller, and instead relied on the derivative and integral gains to respond to the maneuver. This also meant that while the HBM with rotational controllers failed to maintain a stable head position during gravity settling (i.e., the head slowly moved rearwards) they managed to respond well to the accelerations during the maneuvers, when the head moved relatively faster, Figure 6. In the tuning phase, the controllers were activated at the same time as the maneuver initiation, thus the controllers were not tuned to allow for gravity only loading. To mitigate this, future optimizations could integrate the gravity settling phase prior to one or both of the maneuvers, to tune the posture maintenance capabilities.

Two of the optimal designs violated the maximum allowed muscle activity constraints (0.99). This was likely due to the combination of a non-linear behavior in the modified activation response and the linear meta-model used to describe the response in the optimization. The muscle activation signal was saturated, which meant that without the introduced modifications, the optimizer could not differentiate between a model that saturated the signal only briefly or one with sustained saturation. For this reason, the activation response to the optimizer was modified by adding the duration of the saturation to the response signal. Although this helped the optimizer differentiate between the simulations where activity was briefly saturated and simulations where the signal was saturated for longer periods, it introduced non-linearities in the response, because for non-saturated responses it described the muscle activity while after saturation it instead described a duration. For optimizations where the optimal non-saturated response was well below the saturation level, this was not a problem. However, it made it difficult for the algorithm in the cases where it needed to balance the maximum activity constraint and the optimal response, such as the rotational controller tuned to match rotations. One reason for the rotational controller needing maximum activity while the translational controller could respond with sub-maximum activity could be that the rotational controller used relied on derivative control rather than proportional control, as discussed in the previous section, leading to bursts of activity during acceleration ramp-up and rebound.

For the translational controller, the optimized gains were similar to the gains in the baseline model. These baseline gains were established by optimization (Östh et al., 2015) towards volunteer sled tests in a laboratory setting (Ejima et al., 2008), using a previous version of the SAFER HBM. Since that work, several modifications have been made to the SAFER HBM: the soft tissues of the neck and torso has been updated to a more detailed mesh and considerably softer materials (Pipkorn et al., 2021) and the lumbar and cervical spines have been updated and validated with respect to quasi-static loading (Östh et al., 2020). The muscle element origin/insertions and muscle physical cross-sectional areas, however, remained the same between the models, and as such the lever arms and muscle strength has not been updated.

The model displacements and muscle activities were similar for the different optimal gains, indicating that the controller response was relatively insensitive to changes in the range in which the different optima were found (at around 1.2–1.5 1/rad for the proportional gain, and 400–500 ms/rad for the derivative gain). This could also explain why all optimizations used the maximum number of iterations (10), because the difference between the different gain optima identified was much larger (∼20%) compared to the design change limit of 1%, even though the resulting displacements were similar. If the optimizer finds an area in which the response is very similar for different values of the parameters, it becomes difficult to find the global optimum, because all responses are essentially the same. All three translational optimizations yielded similar gains and subsequently similar kinematics and muscle activity, indicating that a similar activation was needed to match both rotations and translations.

All optimization loops were terminated by an iteration limit of 10 and not the design change limit of 1%. This implies that the gains were not fully converged at optimization termination. Based on the convergence plots, Supplementary Figure S5, it is possible that the gains would have changed slightly if more iterations had been performed. As discussed previously, based on the difference between translational models, Figures 5, 8, the resulting predictions would have likely been similar for fully converged optimizations for the translational model. For the rotational controllers the differences between results were larger, Figures 5, 8, thus it is possible that the rotational controller predictions would have been improved with more iterations. However, it is unlikely that more iterations would have changed the rotational controllers from derivative driven to proportional driven, because the proportional gain was decreased in the final iterations for all three rotational controller optimizations, Supplementary Figure S5.

The implementation of the rotational controller was based on a rotation matrix that was then converted to an axis-angle representation. When using projected angles, as was done in the current implementation, this is an extra step that could be bypassed. The formulation was kept in the controller even though it was not needed, because it allows for a more flexible selection of rotations to base the controller on. For instance, the rotation matrix could be reformulated to correspond to Euler angles instead, if there is a need for it in the future.

For most of the tuned controllers in this study, the optimizer kept the integrational part in the controller, while for previous models it was excluded by design. The integral part of the PID controller reduces steady-state error (Åström and Murray, 2021). For instance, in the braking case, integral control would increase the muscle activity during the steady state braking (between approximately 0.5 s and 1.8 s in Supplementary Figure S4), to decrease the forward displacement of the model continuously during steady-state braking. If such behavior was observed in the volunteer responses, integral control would have been appropriate. In the braking maneuver used for gain tuning in this study, Supplementary Figure S4, the forward displacement was somewhat reduced (approx. 10 mm) during the maneuver, which could explain why integral control was kept in most optimization. However, in the braking maneuver used for evaluation simulations, no such behavior was seen (Ólafsdóttir et al., 2013). Instead, the volunteers settled at a displacement level and stayed at a similar displacement level during the steady-state acceleration.

The intention with the controllers was to emulate the VCR, because it was seen in a previous study (Larsson et al., 2019) that emulating VCR gave better kinematic predictions compared to emulating muscle stretch reflexes. In reality, the occupant would most likely use multiple sensory input to guide the muscle activity. For instance, the volunteer experiments were performed with eyes open (Ólafsdóttir et al., 2013; Larsson et al., 2022), and thus the volunteers could have used visual input to regulate their response, rather than vestibular input. In addition, humans can sense and respond to muscle lengthening sensed by muscle spindles (Binder et al., 2009b) and muscle forces sensed by Golgi tendon organs (Binder et al., 2009a), which was not included in the muscle controller. Further, the implemented controllers represent a simplified emulation of the vestibular system reflexes, responding to selected parts of sensory input (two rotations for translational controller and three rotations for rotational controller), while humans sense translational and rotational accelerations of the head in space, and head orientation relative the gravity field (Marieb and Hoehn, 2019). Thus, the controllers represent a simplified model of human response to horizontal plane vehicle maneuvers and are not intended to be direct analogies of human reflexes.

In both control systems, no consideration was taken to the frequency of the input, although the vestibular system has been found to respond differently to input at different frequencies (Cullen, 2019). The controllers were developed to respond in a human-like way to horizontal plane maneuvers, with relatively low frequency loading. The control system might need to be re-tuned or updated if the model should predict the response to higher frequency loading, as has been done with the MADYMO model (Mirakhorlo et al., 2022).

The gains are most likely model specific, such that if the same controller is to be used in another HBM, the gains would need to be re-tuned to that specific HBM. Furthermore, if the controllers are to be used for other maneuvers, or if the SAFER HBM is updated in a way that would influence the response in evasive maneuvers, the controller gains might need to be re-tuned.

The model was not validated after gain tuning. The bio-fidelity of the HBM with tuned controllers was evaluated against volunteer displacements and muscle activity in either the same or similar environments. An important next step would be to validate the HBM in different environments. For example, the model should be validated in horizontal-plane evasive maneuvers by comparing HBM displacements to volunteer displacements as presented in for instance (Kirschbichler et al., 2014) or (Reed et al., 2019). A limitation in this study was that data from three different studies were combined to build the STPs. In the three studies, different number of volunteers were used, different signal normalization techniques were used, and different types of loading were used. To overcome this limitation, the STPs were normalized using maximum activity in each direction. However, when normalizing in this way, the difference between a specific muscle’s activity depending on direction was lost. For instance, before normalizing with direction, sternohyoid (STH) showed lower activity in extension compared to extension combined with left lateral bending (Ólafsdóttir et al., 2015), while after normalized to direction, sternohyoid had equal activity in both extension and extension combined with left lateral bending because sternohyoid was the muscle with highest activity in these two directions. Thus, sternohyoid activity was used to normalize all muscles for these directions. This means that while the volunteers would activate sternohyoid slightly less when going from extension and left lateral bend to only extension, the model would instead activate all other muscles slightly more.

There were also some limitations relating to the data used as input to the STPs. The STP used for the flexion/extension and lateral bending was originally created with respect to acceleration direction (Ólafsdóttir et al., 2015) and assumed to correlate with head displacement in the opposite direction in the translational controller (Ólafsdóttir et al., 2019). When transferring these spatial patterns to the rotational controller, the STPs were instead assumed to correlate to the direction of rotation and to include only flexion/extension and lateral bending. However, these assumptions could not be confirmed because no video analysis was performed. The two additional experiments, where axial rotation was included, include only two subjects. In addition, one of these experiments include isometric data, and it has previously been shown that dynamic and isometric STPs can differ (Siegmund et al., 2007). The muscle activity in these two experiments were recorded bilaterally. Muscle activity from both left and right sides were used when calculating average activity for each muscle and direction. The left muscle activity was mirrored (by flipping horizontal angle and axial rotation angle in Supplementary Table S2) to create additional “right side” muscle activity. It is unclear if this assumption of symmetry is valid. A visual comparison between left-side and right-side muscle activity was done prior to averaging across both sides, and activities were similar between the two sides after mirroring, but no objective evaluation was done. Since only two subjects were included, it is possible that the method of mirroring does not generalize to a larger population. A further limitation is that the two experiments containing axial rotation were normalized using peak muscle activity within each experiment, and not MVIC. This was done because the experiments were performed as pilot testing prior to other experiments, and not specifically as input for muscle controllers. To mitigate this problem, the experiments were ranked in priority, but non-MVIC normalized data was still used for directions where no other data was available. Similar to the translational controller not responding to rotations, the rotational controller does not respond to pure protraction/retraction. Humans can protract/retract without rotating the head (Carlsson et al., 2021), and this movement would be invisible to the controller. In the current study, this was not found to cause problems with the controller, but if the model would protract without rotating the head in another loading condition, one additional controller responding to protraction/retraction could be added on top of the rotational controller.

## 5 Conclusion

The results show that when tuned, both the translational and rotational controllers can be used to predict the occupant response to an evasive maneuver, allowing for the inclusion of evasive maneuvers prior to a crash in evaluation of vehicle safety. The rotational controller could predict omni-directional head displacements with fair to good bio-fidelity, but the translational controller outperformed the rotational controller. Thus, for now, the recommendation is to use the translational controller with tuned gains.

## Data Availability

The original contributions presented in the study are included in the article/Supplementary Material, further inquiries can be directed to the corresponding author.
