# Supplementary material for: Predicting occupant head displacements in evasive maneuvers; tuning and comparison of a rotational based and a translational based neck muscle controller
Source: Front Bioeng Biotechnol. 2024 Jan 12;11:1313543. doi: 10.3389/fbioe.2023.1313543 (PMC10811264; doi:10.3389/fbioe.2023.1313543)
Supplement: Supplementary file 1 [file DataSheet1.docx]

Supplementary Material

| Table S1. CORA settings and rating limits | | | | | |
| --- | --- | --- | --- | --- | --- |
|  |  | Weight - sub-system evaluation | Weight -gain evaluation |  | Weight |
| Settings | Corridor Method | 0 | 0.5 | - | - |
|  | Correlation Method | 1 | 0.5 | Cross correlation function | 0.5 |
|  |  |  |  | Size | 0.25 |
|  |  |  |  | Phase shift | 0.25 |
| Limits on CORA rating | Rating | | Limit | | |
|  | Excellent | | >0.94 | | |
|  | Good | | >0.80 | | |
|  | Fair | | >0.58 | | |
|  | Poor | | <=0.58 | | |

## Controller development

| Table S2. Spatial tuning matrix structure, including sources for muscle activity data, Perturbations (P) (Ólafsdóttir et al., 2015), Experiment 1 (dynamic, D) or Experiment 2 (isometric (I)). Direction described in principal directions (axial rotation (AR), lateral bending (LB), flexion (FL) /extension (EX)) and their combinations. For AR and LB, direction is indicated as left (L) or right (R). Direction on upper rows (bold font) and data source in lower rows (italic font) for each axial rotation level. The “no axial rotation” correspond to the STPs used in the translational controller. | | | | | | | | | | |
| --- | --- | --- | --- | --- | --- | --- | --- | --- | --- | --- |
| **Horizontal angle** | | | | | | | | | | |
|  |  | -180 | -135 | -90 | -45 | 0 | 45 | 90 | 135 | 180 |
| **Axial rotation** | Pure axial rotation - left | **AR L** | | | | | | | | |
|  |  | *D* | | | | | | | | |
|  |  | **EX + AR L** | **EX + LB L +AR L** | **LB L + AR L** | **FL + LB L +AR L** | **FL + AR L** | **FL + LB R +AR L** | **LB R + AR L** | **EX + LB R +AR L** | **EX + AR L** |
|  |  | *D* | *I* | *I* | *I* | *D* | *I* | *I* | *I* | *D* |
|  | No axial rotation | **EX** | **EX + LB L** | **LB L** | **FL + LB L** | **FL** | **FL + LB R** | **LB R** | **EX + LB R** | **EX** |
|  |  | *P* | *P* | *P* | *P* | *P* | *P* | *P* | *P* | *P* |
|  |  | **EX + AR R** | **EX + LB L +AR R** | **LB L + AR R** | **FL + LB L +AR R** | **FL + AR R** | **FL + LB R +AR R** | **LB R + AR R** | **EX + LB R +AR R** | **EX + AR R** |
|  |  | *D* | *I* | *I* | *I* | *D* | *I* | *I* | *I* | *D* |
|  | Pure axial rotation - right | **AR R** | | | | | | | | |
|  |  | *D* | | | | | | | | |

| Table S3. Muscle STP groups, including data availability (indicated with X) and substitution if no data available. | | |
| --- | --- | --- |
| Muscle | Perturbations  (Ólafsdóttir et al., 2015) | Experiment 1 and Experiment 2 |
| Sternocleidomastoid  (SCM) | X | X |
| Sternohyoid  (STH) | X | X |
| Levator scapulae  (LS) | X | X |
| Trapezius  (Trap) | X | LS |
| Splenius capitis  (SPL) | SCerv | X |
| Semispinalis capitis  (Scap) | X | X |
| Semispinalis cervicis  (Scerv) | X | SPL |
| Cervical multifidus C4–C5 level  (CM-C4) | X | X |
| Cervical multifidus C6–C7 level  (CM-C6) | X | CM-C4 |
| Occipital capitis inferior  (OCI) | CM-C6 | X |
| Rectus capitis posterior major  (RCP) | SCap | X |

| Table S4. Muscles included in the STP groups for the rotational controller. For muscle groups SPL, OCI and PRC the abbreviations in parenthesis describe the grouping used in the translational controller. In the other muscle groups, muscles belong to the same group for both control systems. | | | | | | | | | | | |
| --- | --- | --- | --- | --- | --- | --- | --- | --- | --- | --- | --- |
| SCM | STH | LS | | Trap | Scap | Scerv | CM-C4 | CM-C6 | SPL | OCI | RCP |
| Sterno-cleido-mastoid | Suprahyoid | Levator scapulae | | Trapezius | Semi-spinalis capitis | Semi-spinalis cervicis | Mfc-C2 C5 | Mfc-C3 C7 | Splenius-capitis (SCerv) | Obliq.- capitis inferior (CM-C6) | Rectus capitis post. minor (SCap) |
| Scalenus posterior | Sternohyoid | | |  | Obliqus capitis superior | Semi-spinalis thoracis | Mfc-C2 C6 | Mfc-C4C7 | |  | Rectus capitis post. major (SCap) |
| Scalenus medius | Sternothyroid | | |  |  | Splen-ius cervicis | Mfc-C3 C6 | Mfc-C4T1 | |  |  |
| Scalenus anterior | Omohyoid | | |  |  | Erector spinae longissimus capitis | | Mfc-C5T1 | |  |  |
| Rectus capitis anterior | Longus colli superior oblique | | | | | Erector spinae longissimus cervicis | | Mfc-C5T2 | |  |  |
|  | Longus colli vertical | | | |  | Erector spinae iliocostalis cervicis | | Mfc-C6T2 | |  |  |
|  | Longus colli inferior oblique | | | | |  |  | Mfc-C6T3 | |  |  |
|  | Longus capitis | | |  |  |  |  | Mfc-C7T3 | |  |  |
|  |  | |  |  |  |  |  | Mfc-C7T4 | |  |  |
|  |  | |  |  |  |  |  | Rectus capitis lateralis | | |  |

$$\boldsymbol{R}=\left[ \begin{matrix} cos(r_{y})cos(r_{z}) & -cos(r_{y})sin(r_{z}) & sin(r_{y}) \\ cos(r_{x})sin(r_{z}) + cos(r_{z})sin(r_{x})sin(r_{y}) & cos(r_{x})cos(r_{z}) - sin(r_{x})sin(r_{y})sin(r_{z}) & -cos(r_{y})sin(r_{x}) \\ sin(r_{x})sin(r_{z}) - cos(r_{x})cos(r_{z})sin(r_{y}) & cos(r_{z})sin(r_{x}) + cos(r_{x})sin(r_{y})sin(r_{z}) & cos(r_{x})cos(r_{y}) \end{matrix} \right]$$

Equation S1

$$\boldsymbol{v}=\left[ \begin{matrix} cos(r_{y})sin(r_{x}) + cos(r_{z})sin(r_{x}) + cos(r_{x})sin(r_{y})sin(r_{z}) \\ sin(r_{y}) - sin(r_{x})sin(r_{z}) + cos(r_{x})cos(r_{z})sin(r_{y}) \\ cos(r_{x})sin(r_{z}) + cos(r_{y})sin(r_{z}) + cos(r_{z})sin(r_{x})sin(r_{y}) \end{matrix} \right]$$

Equation S2

$$\theta=\frac{cos(r_{x})cos(r_{y}) + cos(r_{x})cos(r_{z}) + cos(r_{y})cos(r_{z}) - sin(r_{x})sin(r_{y})sin(r_{z})-1}{2}$$

Equation S3

### Spatial tuning patterns

| Table S5. Spatial tuning patters for muscle groups, right side muscles. Each STP is divided into 5 levels, corresponding to rows in Table V. Left- and rightmost show muscle activity when returning to neutral position from a pure axial rotational displacement, left on left side (for a right-side muscle this would mean activity to produce ipsilateral rotation, to return back to neutral position), right on right side (for a right-side muscle this would mean muscle activity to produce contralateral rotation, to return back to neutral position). The three center polar plots show the STP of flexion/extension and lateral bending, with three diferent levels of axial rotation (left axial rotation, no axial rotation, right axial rotation, from left-hand-side to right-hand-side).  The flexion (up)/extension (down) and left (left) and right (right) lateral bending is indicated by the angle in the polar plot. Since the pure axial rotations contain no flexion/extension or lateral bending, these consist of a single value, but for easier visual interpretation and comparison the pure axial rotations were presented as circles in polar plots.  In the first plot, an example STP is used, with directions visualized for flexion/extension (green), lateral bending (blue) and axial rotation (grey). One example point is shown in orange, with right axial rotation and flexion, without lateral bending.  The second plot shows the combinations for the five different polar plots. From the third plot, STPs used in rotational controller are shown. |
| --- |
| Example, how to read the plots (Sternocleidomastoid (SCM))  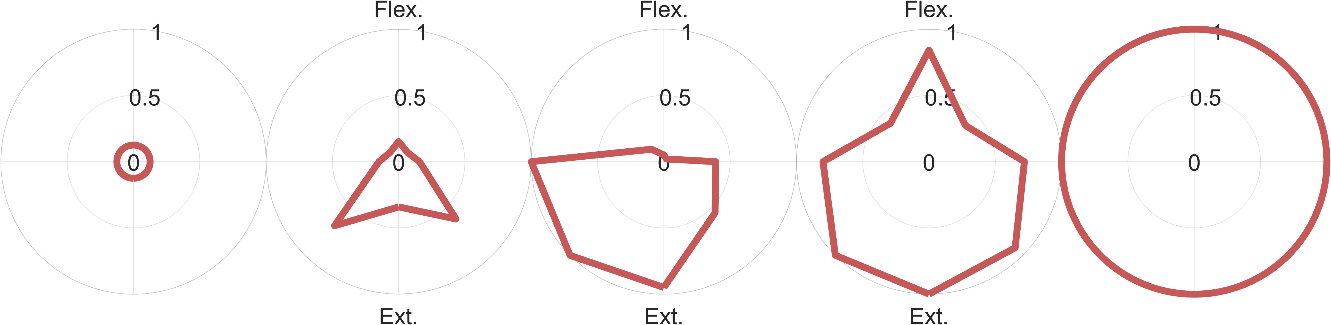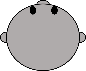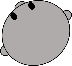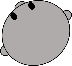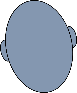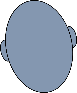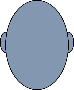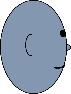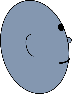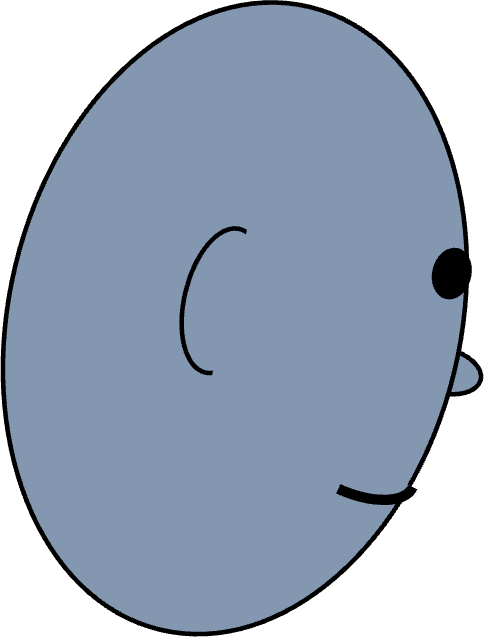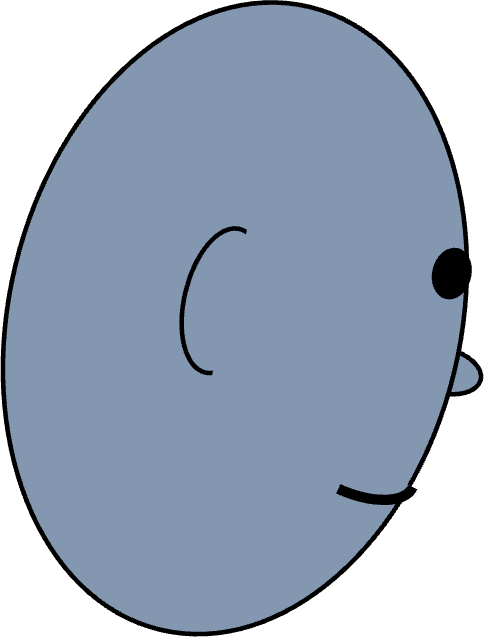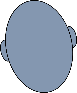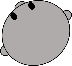 |
| 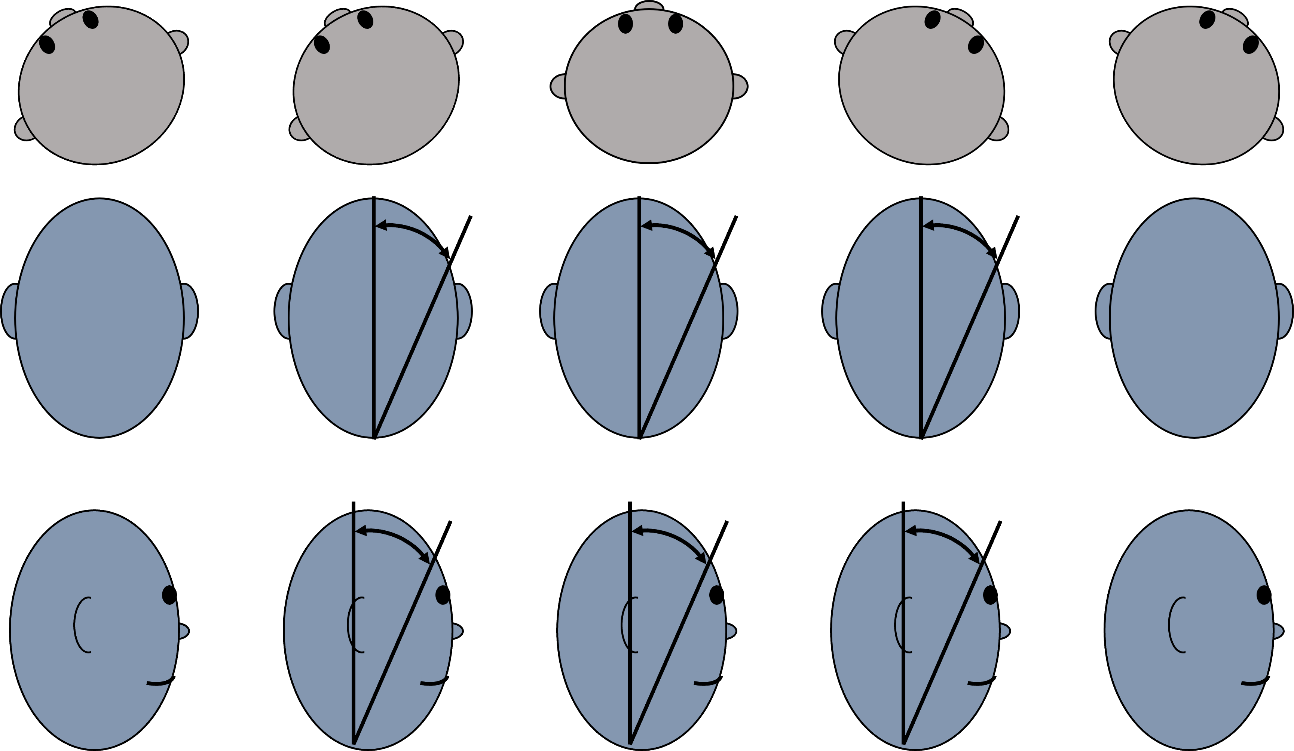 |
| Sternocleidomastoid (SCM)  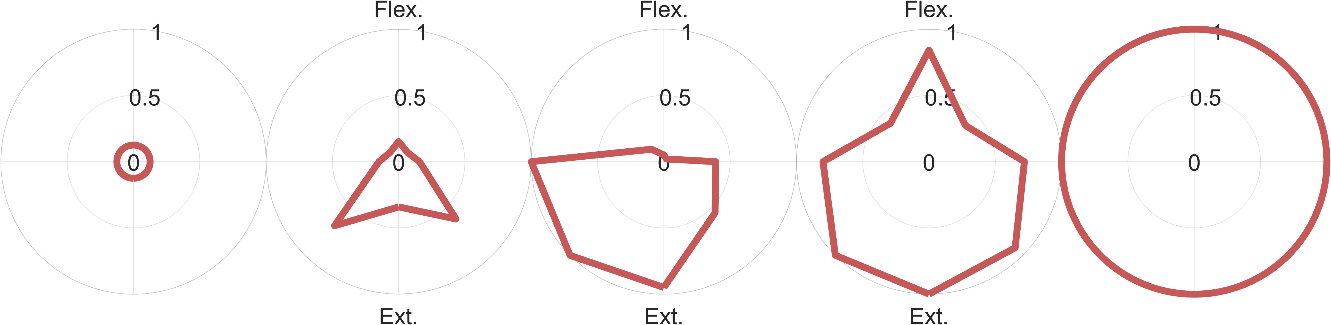 |
| Sternohyoid (STH)  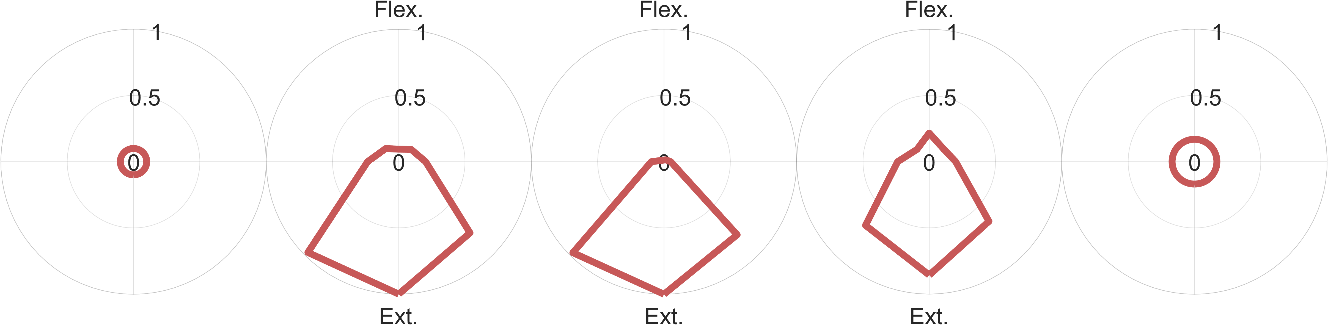 |
| Levator scapulae (LS)  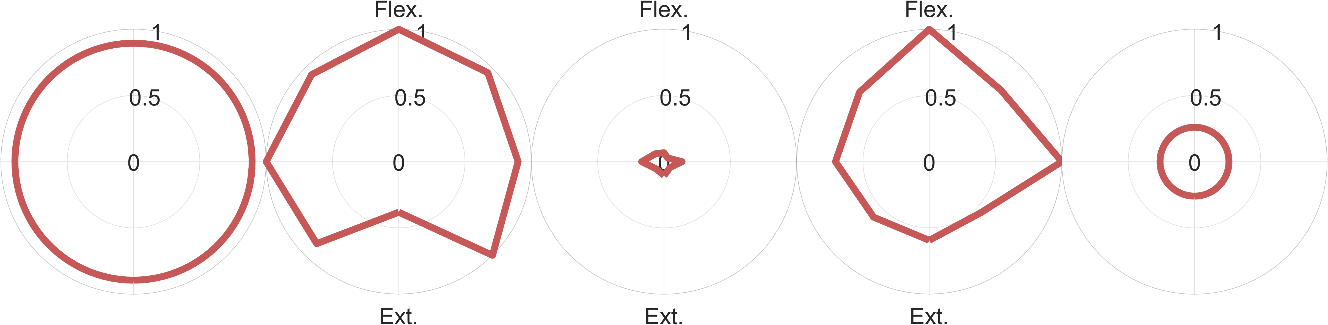 |
| Trapezius (Trap)  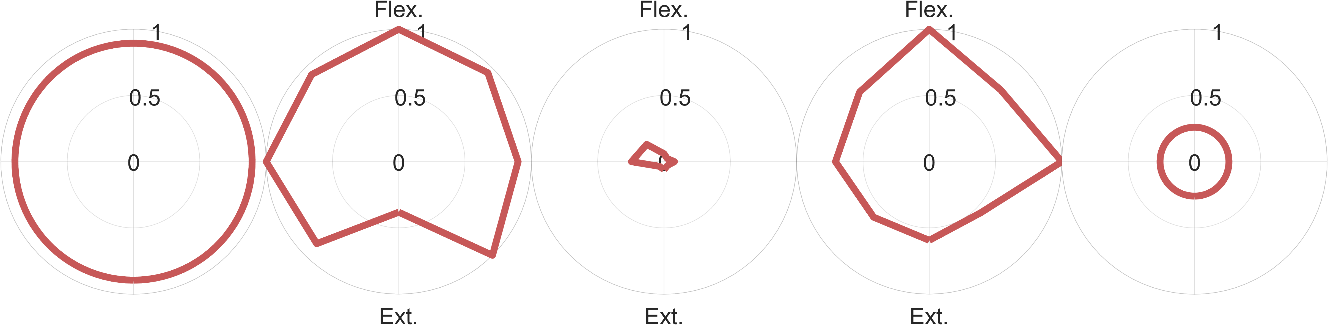 |
| Splenius capitis (SPL)  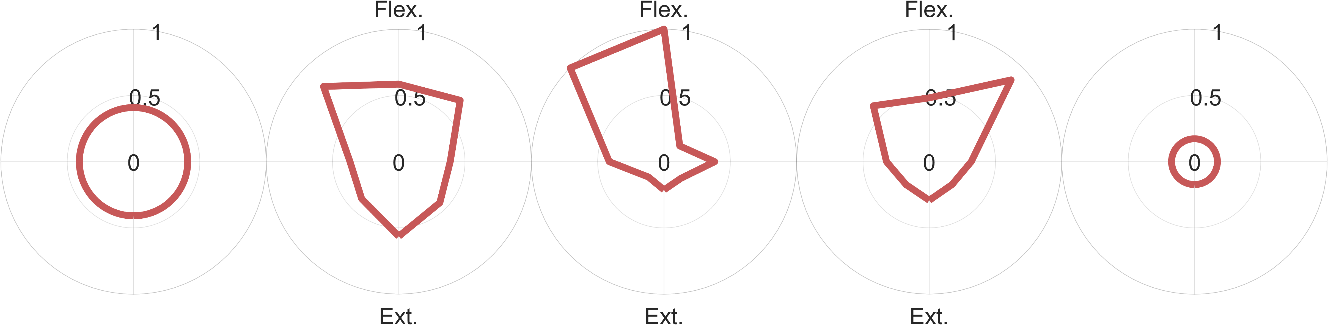 |
| Semispinalis capitis (SCap)  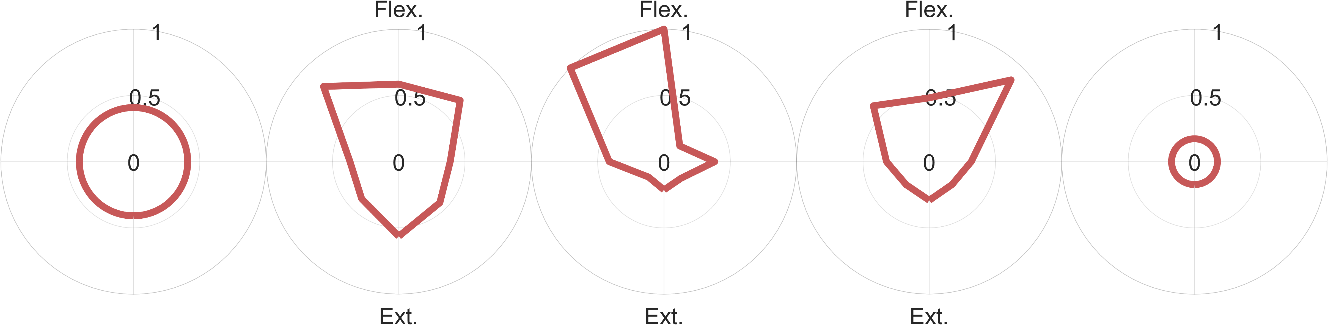 |
| Semispinalis cervicis (SCerv)  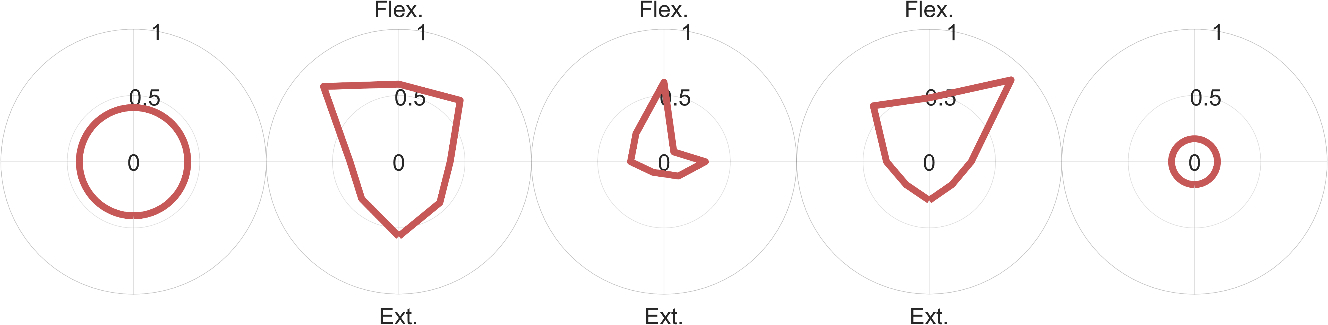 |
| Cervical multifidus C4–C5 level (CM-C4)  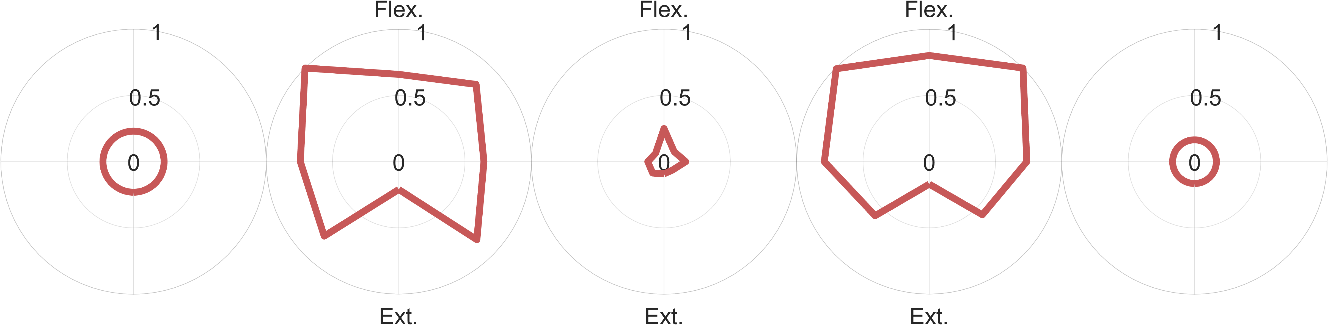 |
| Cervical multifidus C6–C7 level (CM-C6)  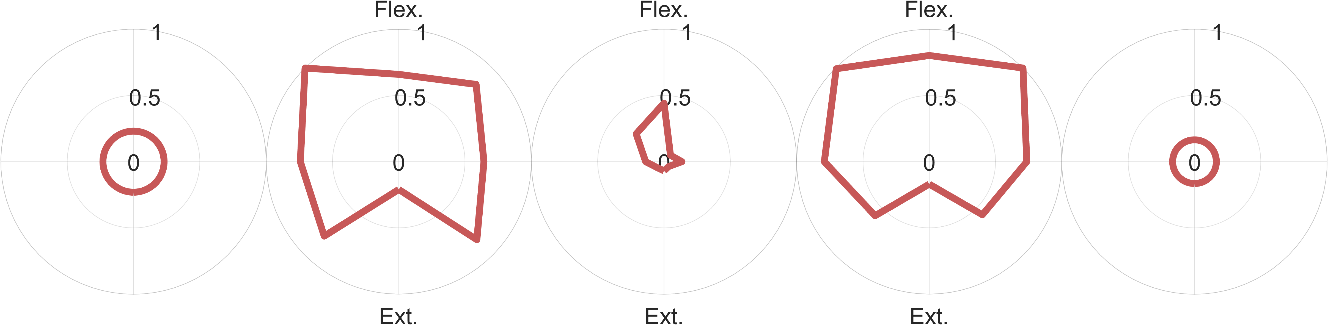 |
| Occipital capitis inferior (OCI)  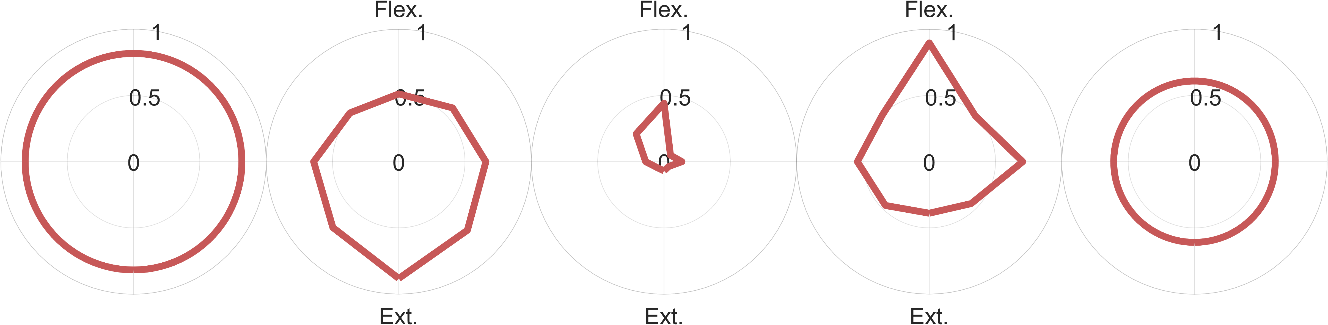 |
| Rectus capitis posterior major (RCP)  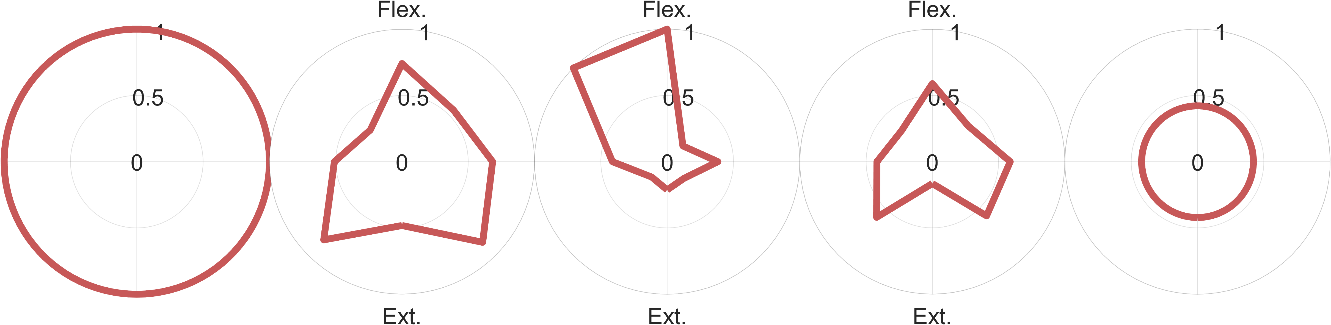 |

### Available spatial tuning data

The available data were visualized in figures in Table S6, prior to any re-normalization described in the publication, section 2.3.3 Combining data. In the plots below, the activity from Perturbation data were normalized first by MVIC values, and after that by the maximum value of any muscle and any direction (SCap, flexion + right lateral bending, which in the figure has an activity of 1). The additional experiments were normalized with peak response during the experiment, as described in the manuscript under section 2.3.2.2 Additional experiments, again, prior to the re-normalization described in section 2.3.3 Combining data. For some muscles, such as trapezius, no data was available for certain data points, substitute patterns were assigned according to Table S3.

| Table S6. Bar graphs with available data, following the structure described in Table S2, with sources: Experiment 1 (dynamic, D), Experiment 2 (isometric (I)) or Perturbations (P) (Ólafsdóttir et al., 2015). Direction described in principal directions (axial rotation (AR), lateral bending (LB), flexion (FL) /extension (EX)) and their combinations. For AR and LB, direction is indicated as left (L) or right (R). Each row describes a level of axial rotation, while flexion/extension and lateral bending is described by the columns. |
| --- |

| Sternocleidomastoid (SCM)  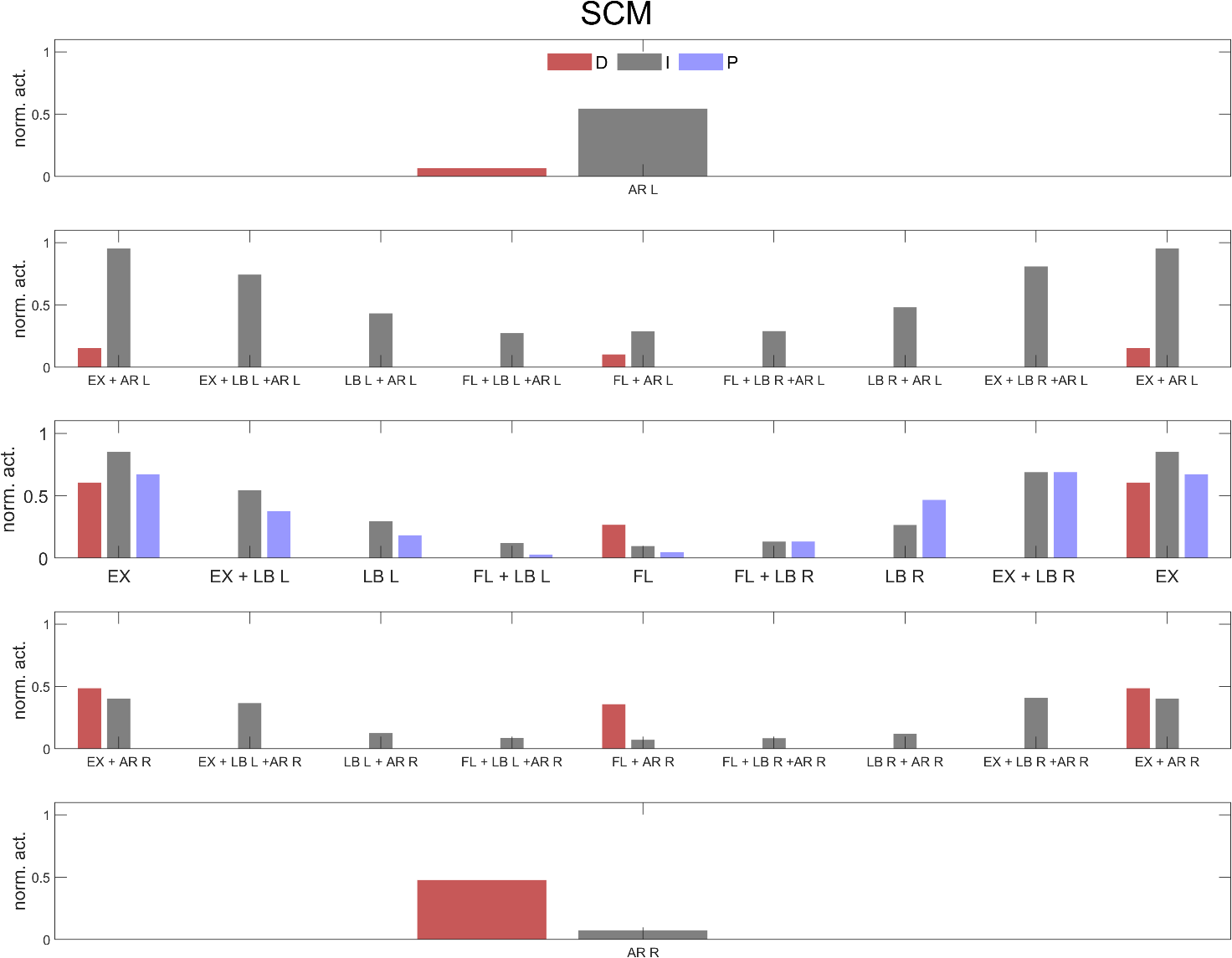 |
| --- |

| Sternohyoid (STH)  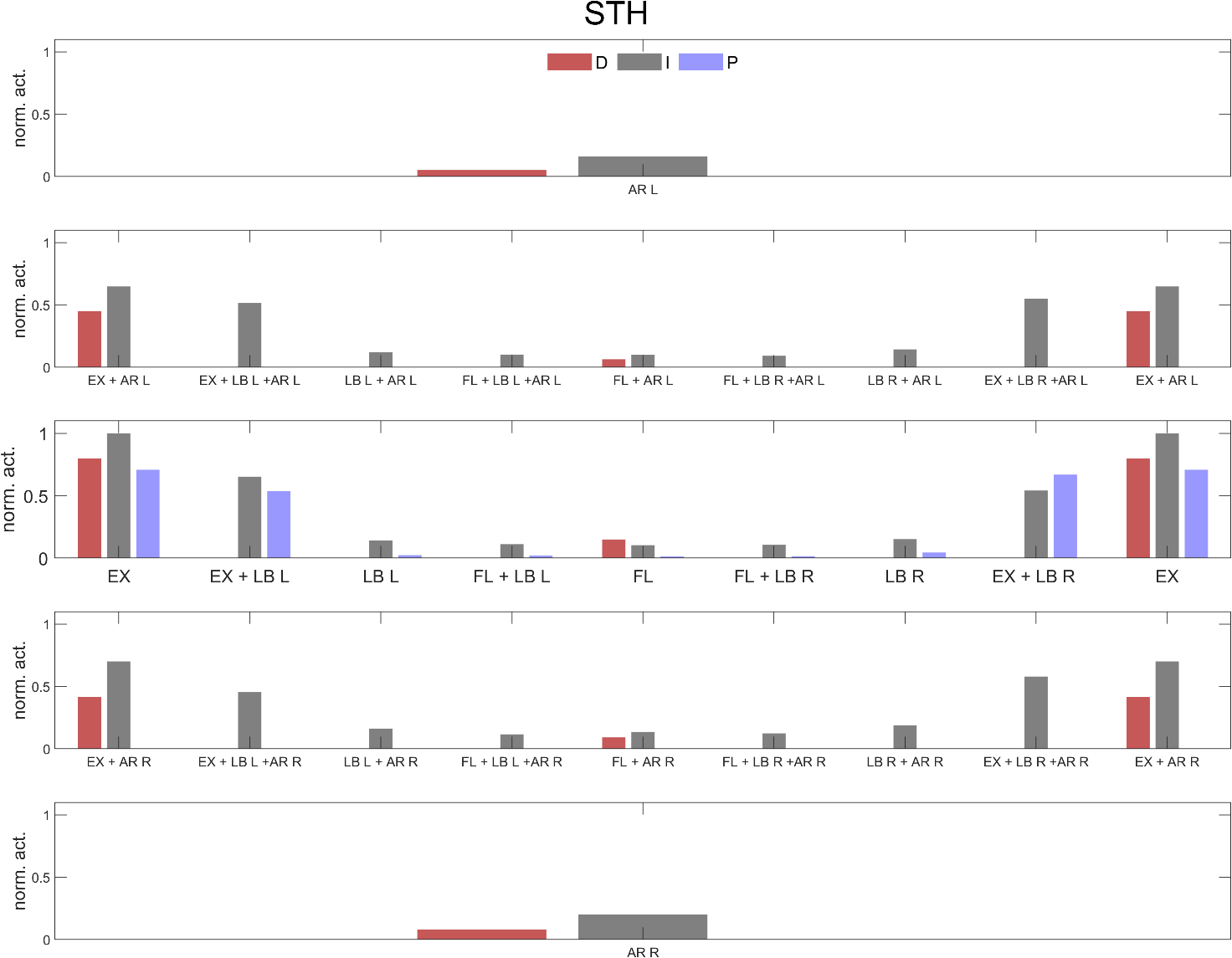 |
| --- |

| Levator scapulae (LS)  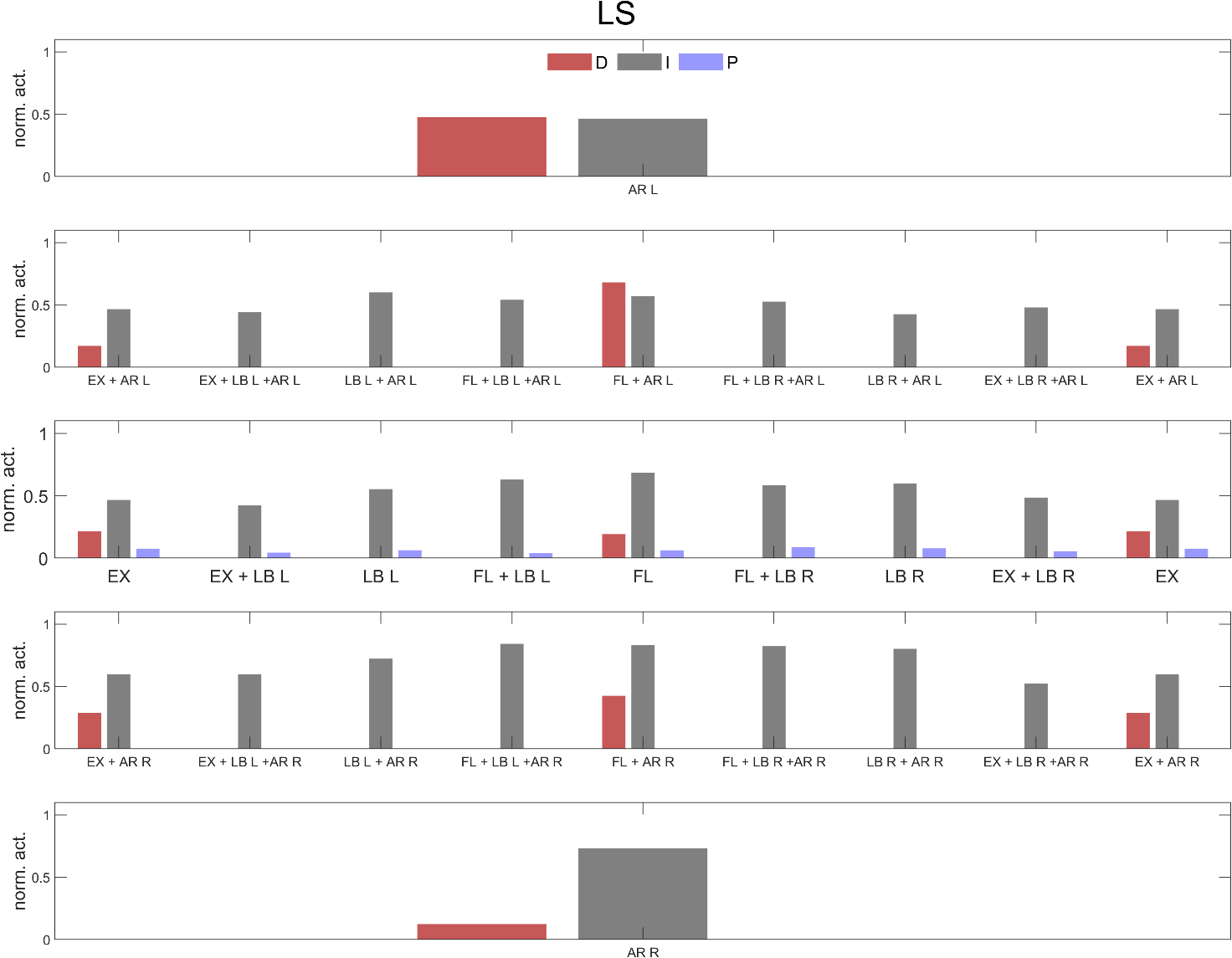 |
| --- |

| Trapezius (Trap)  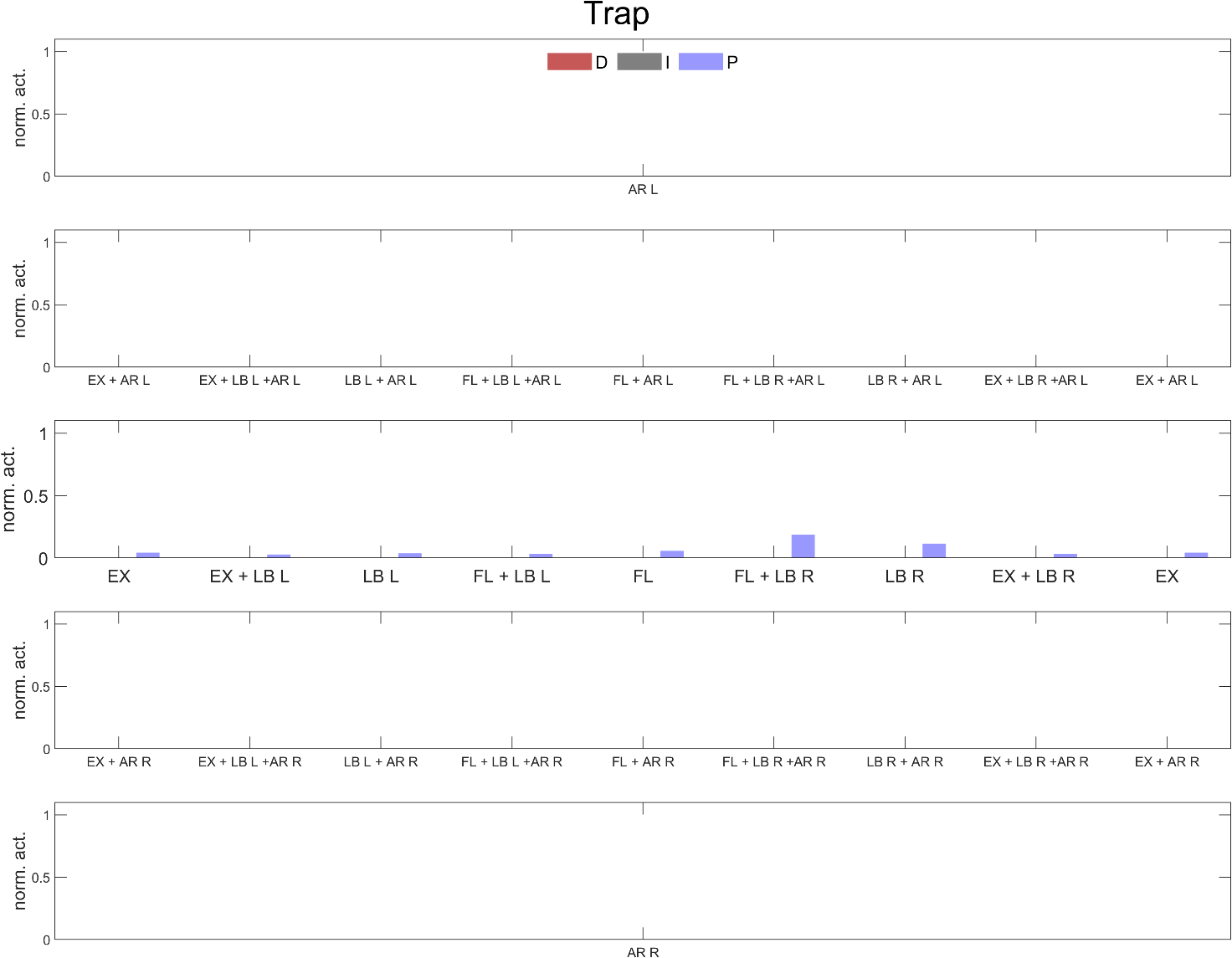 |
| --- |

| Splenius capitis (SPL)  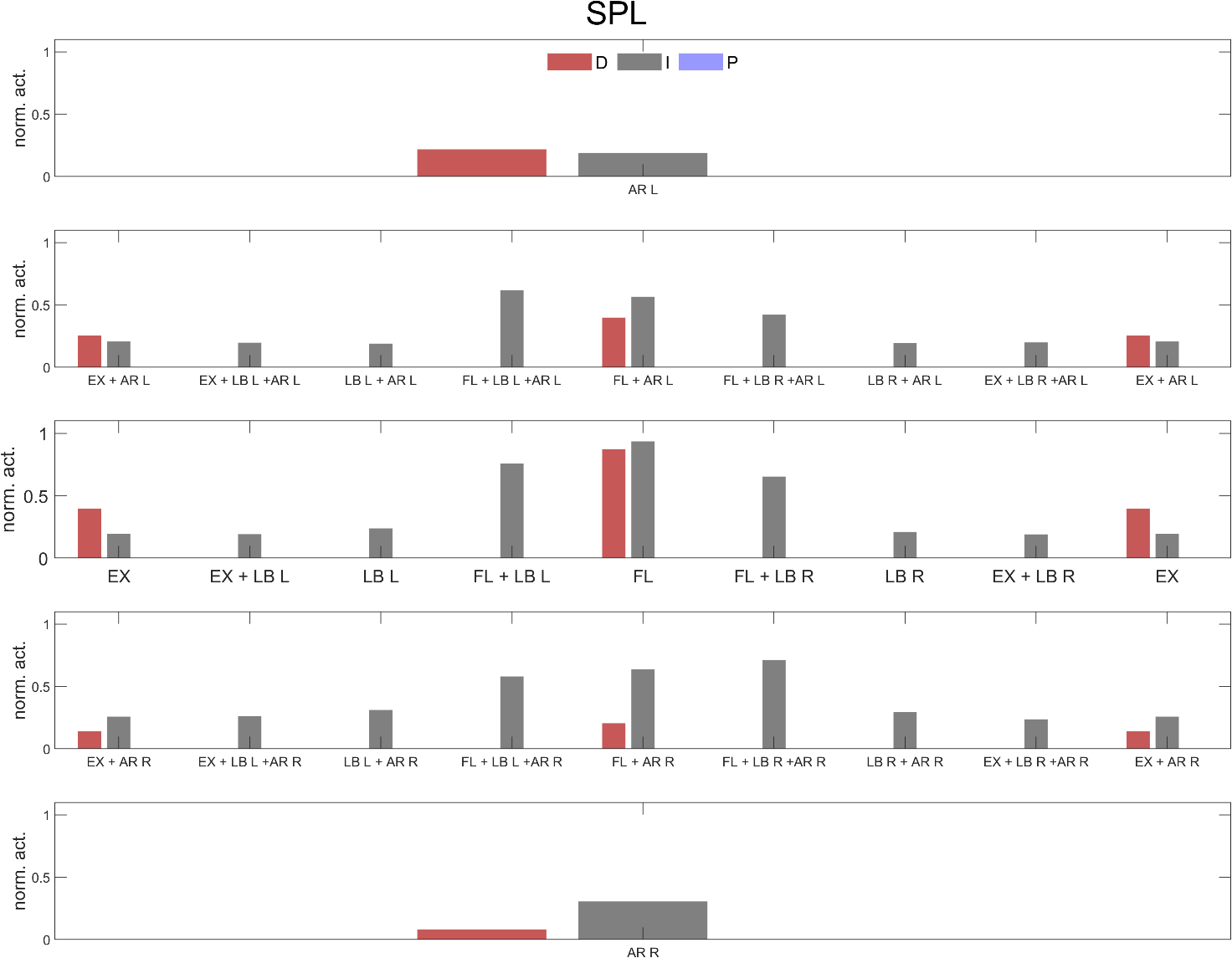 |
| --- |

| Semispinalis capitis (SCap)  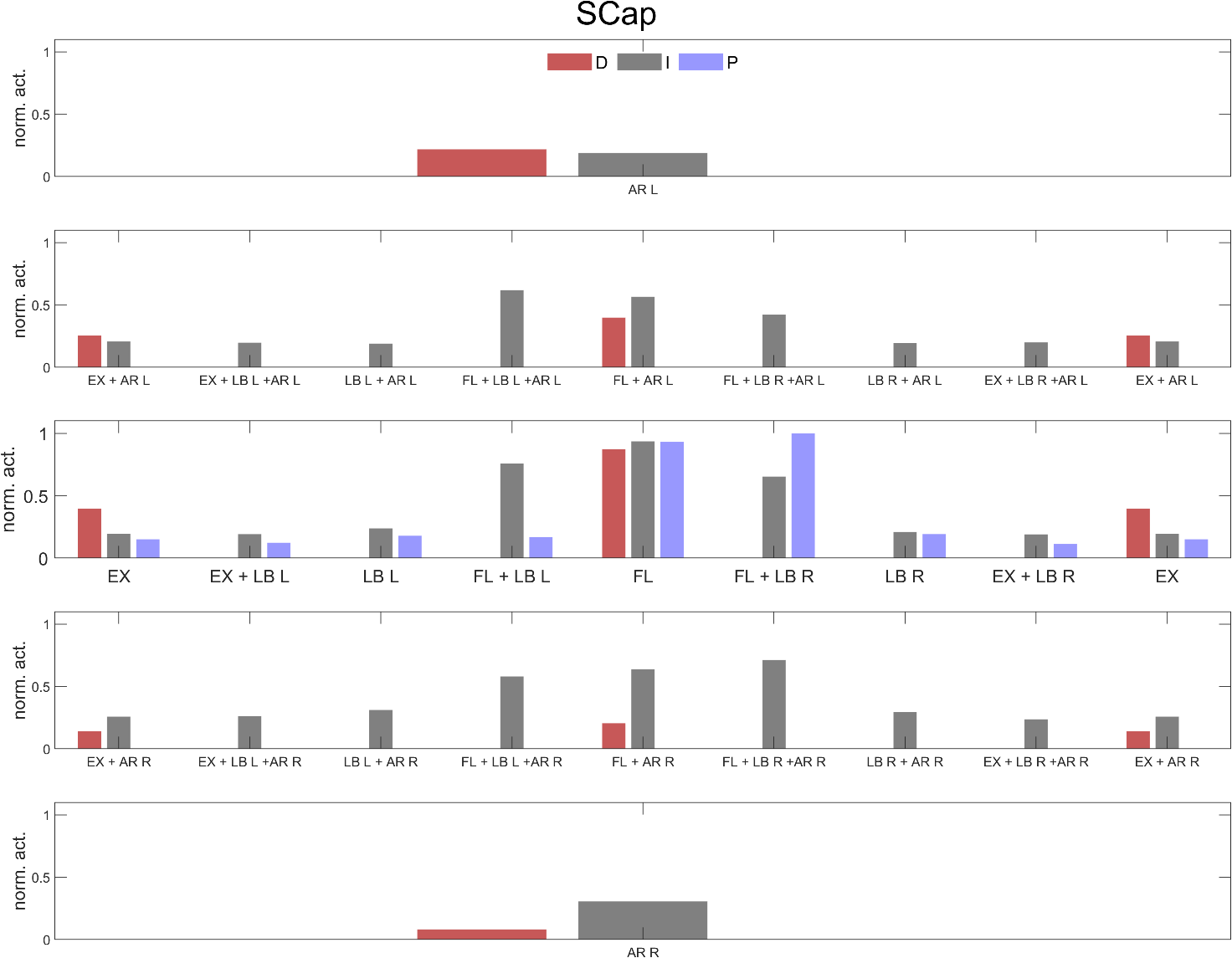 |
| --- |

| Semispinalis cervicis (SCerv)  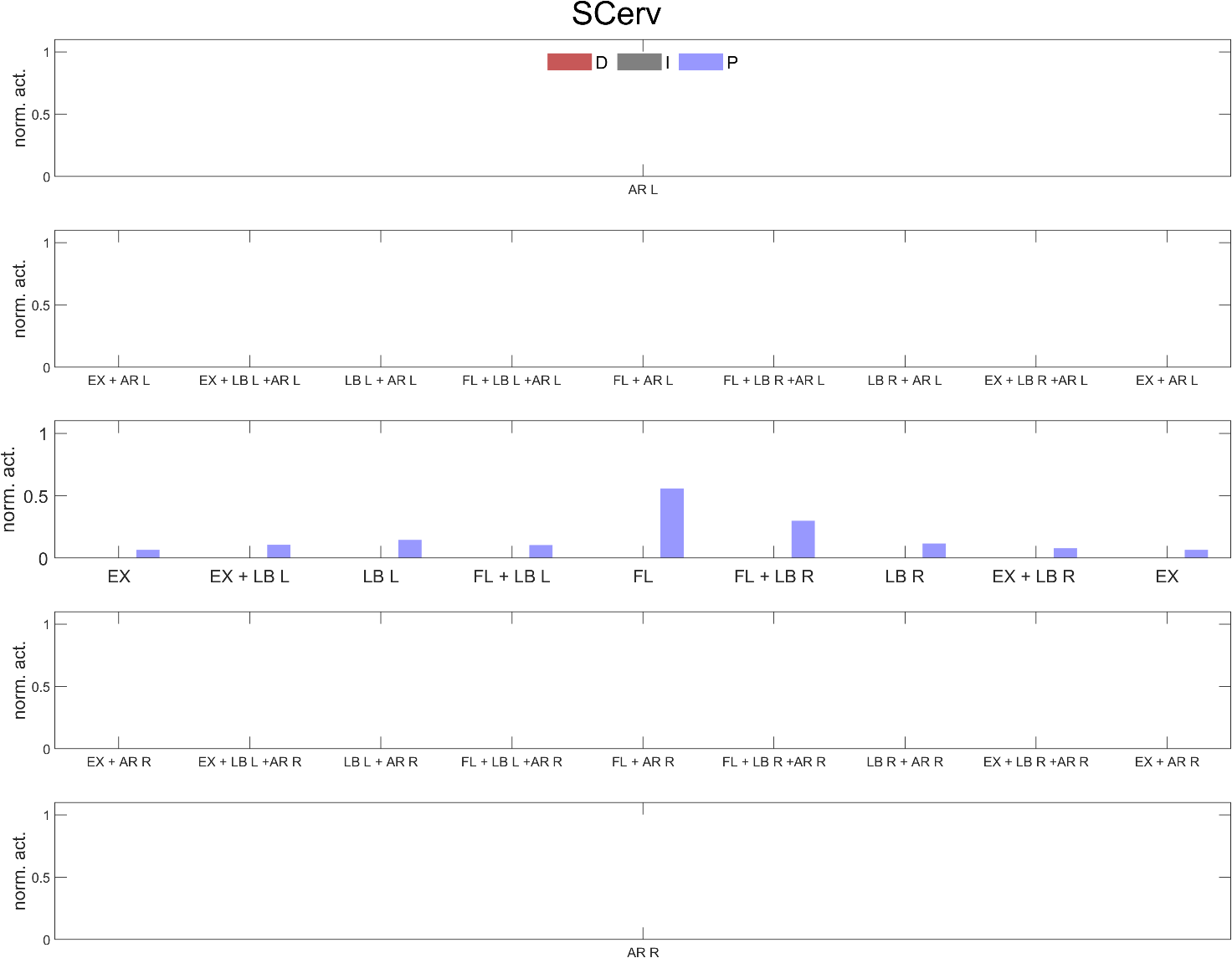 |
| --- |

| Cervical multifidus C4–C5 level (CM-C4)  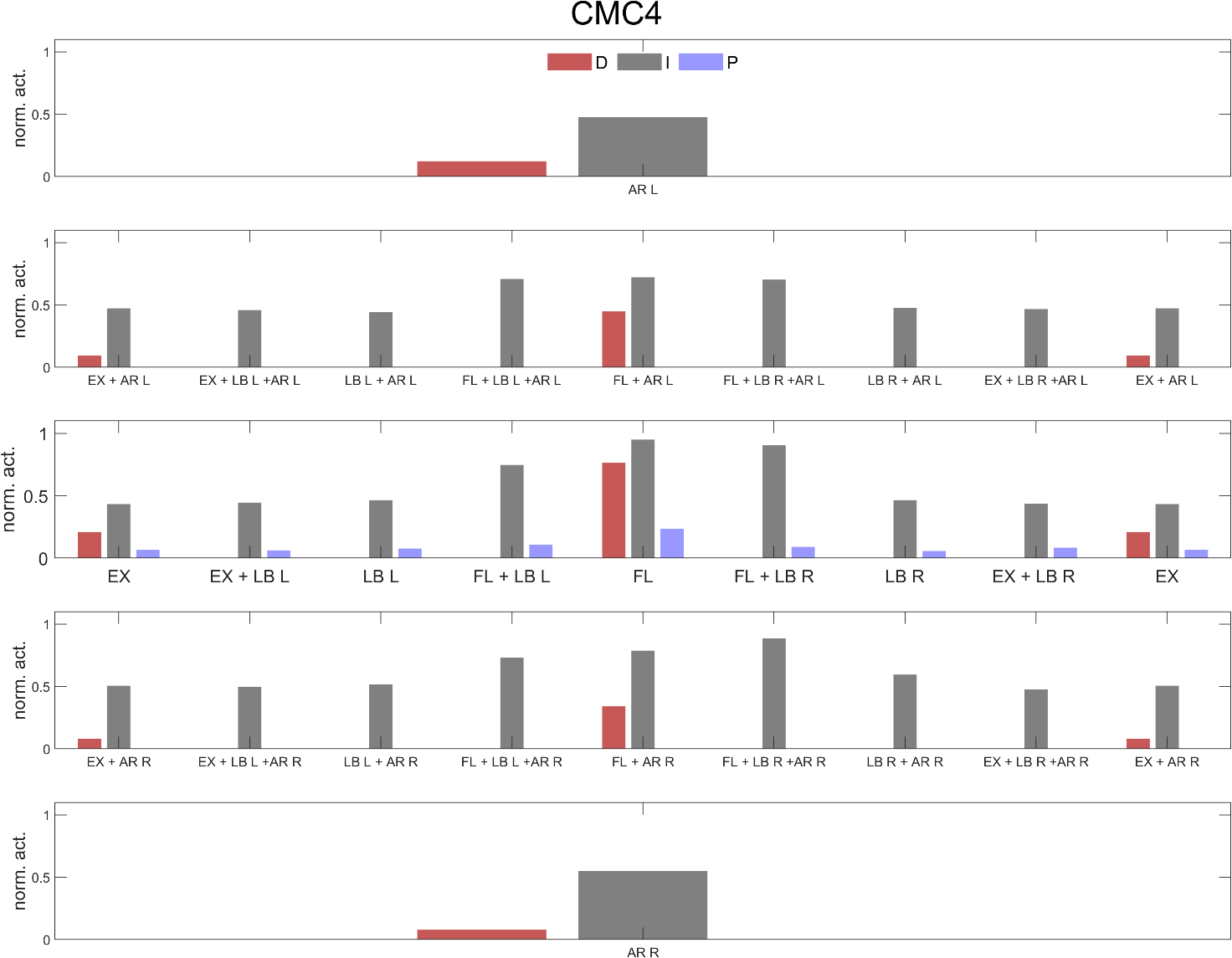 |
| --- |

| Cervical multifidus C6–C7 level (CM-C6)  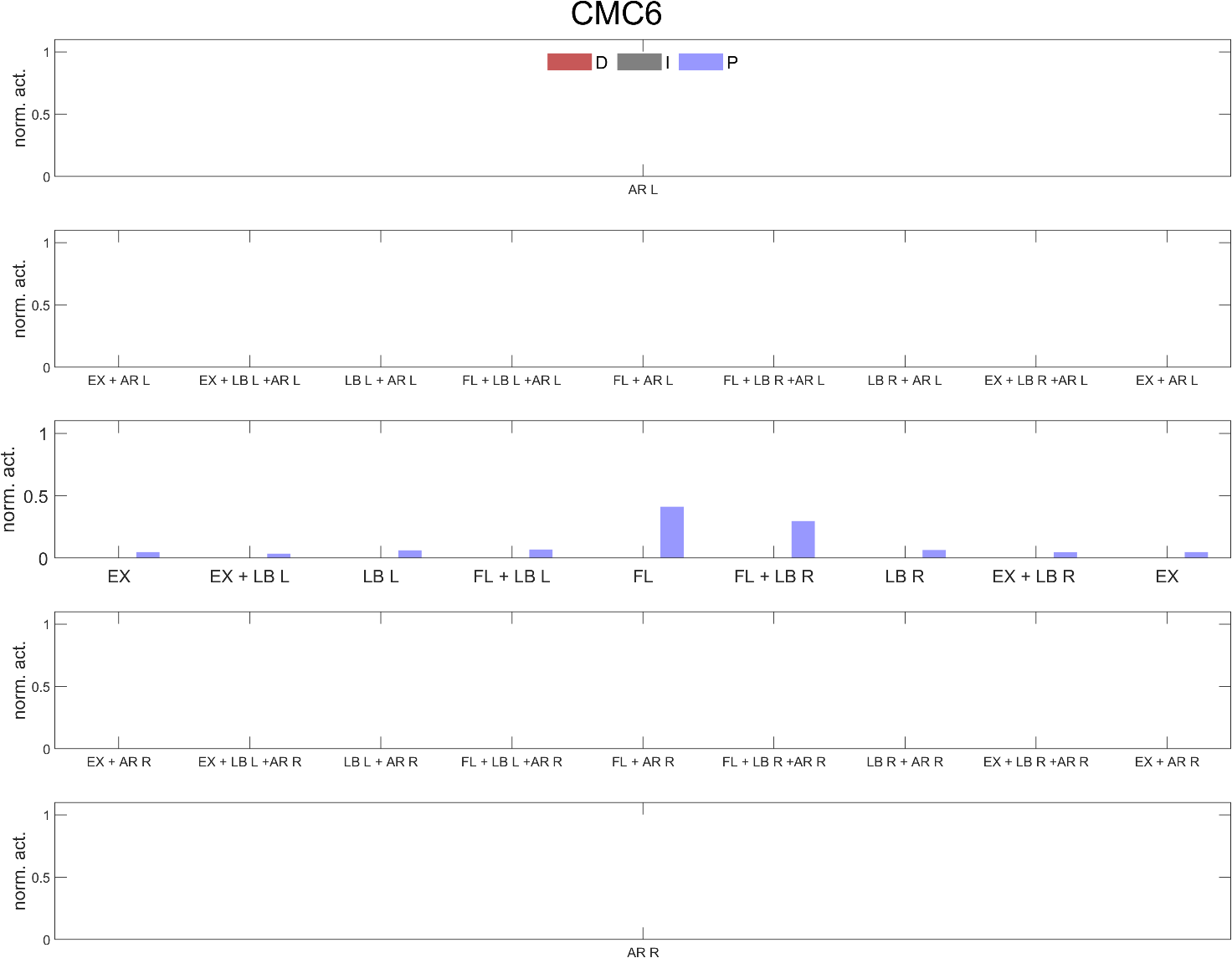 |
| --- |

| Occipital capitis inferior (OCI)  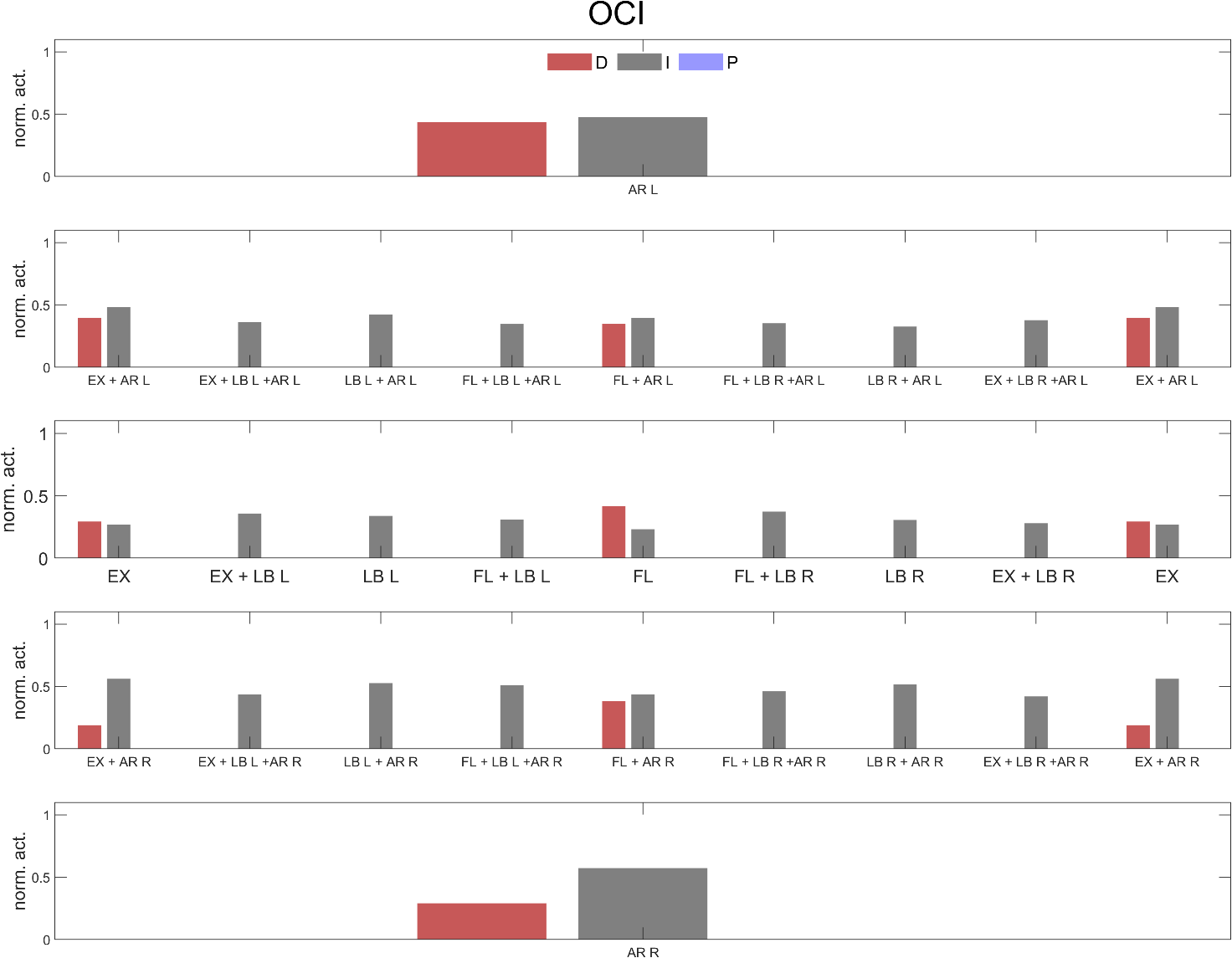 |
| --- |

| Rectus capitis posterior major (RCP)  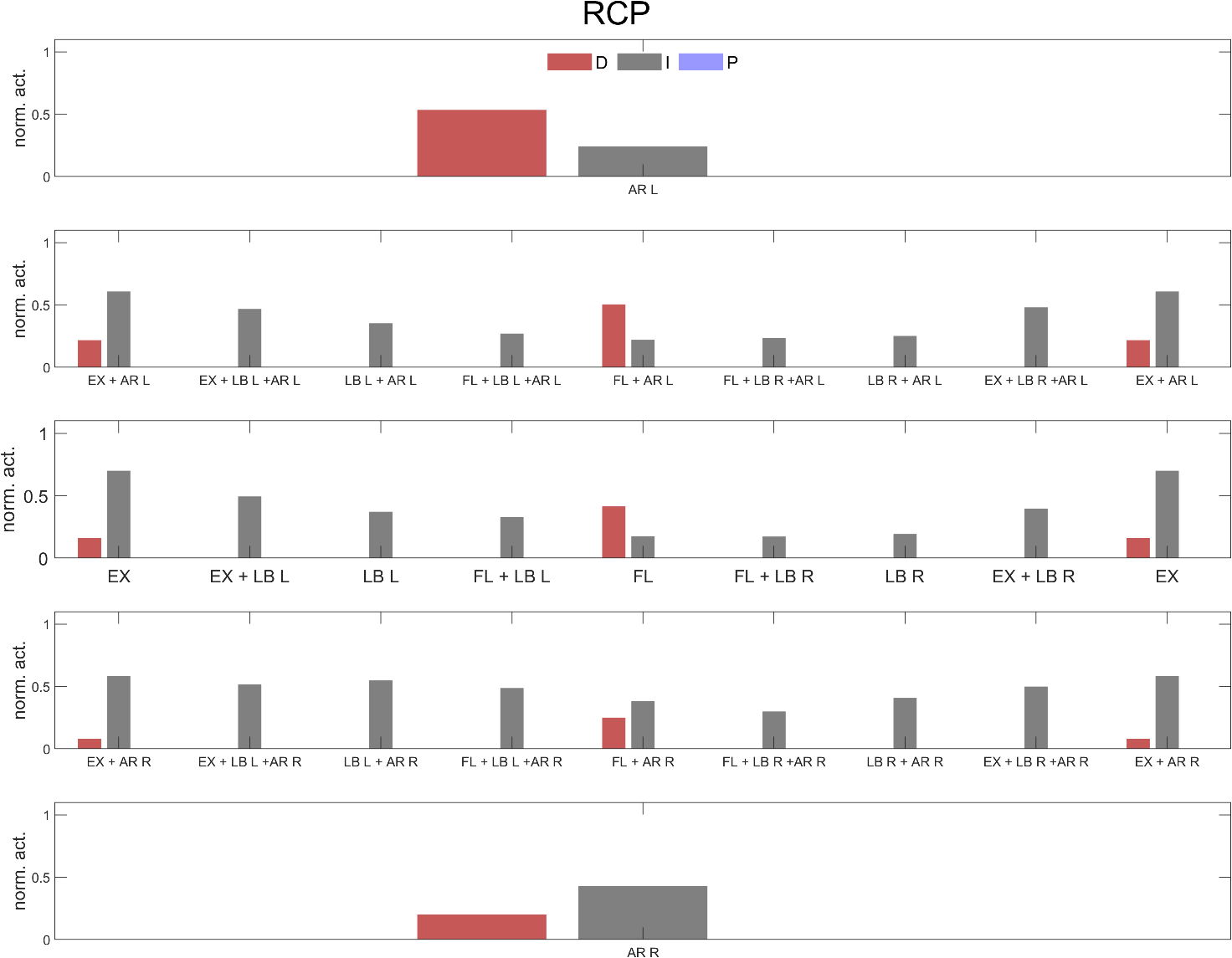 |
| --- |

## Sub-system evaluation

For a braking maneuver, Figure S1 shows that the translations and rotations of the sub-system were similar to that of the full model, with a maximum difference of 15 mm (10 %) in translation (x direction) and 5° (62 %) in rotation (y direction). The sub-system head forward displacement (positive x) during braking (t>0) was slightly larger than for the full model, while the head y rotation during braking was slightly smaller up until the peak value where the models gave the same peak rotation. Prior to braking onset (t<0), the models behaved similarly, with a small difference (2 mm) seen in head y rotation.


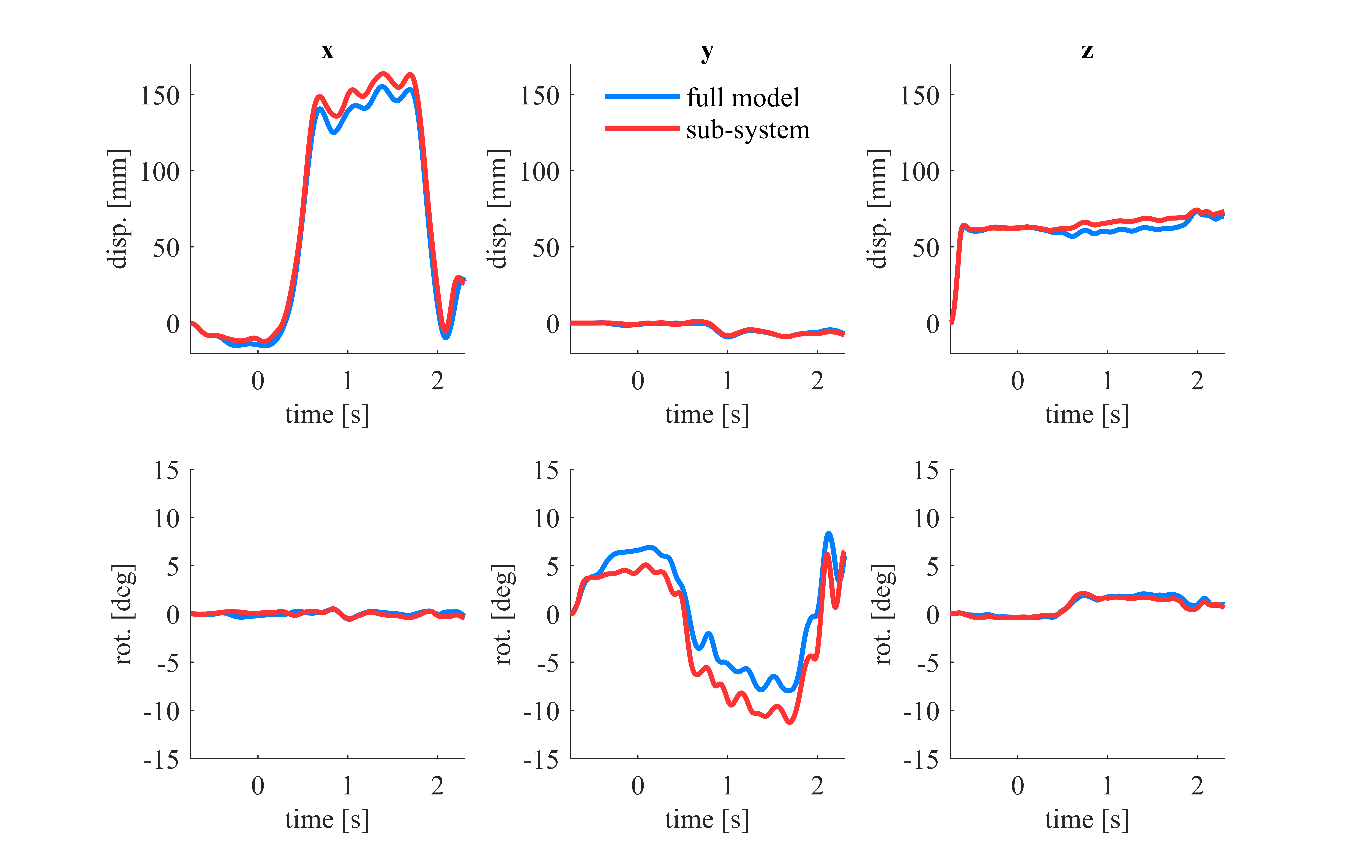


Figure S1. Comparison of head kinematics for full model (blue) and sub-system (red) in braking simulations.

In lane change, Figure S2, the models were also similar, but compared to breaking the difference between the models was slightly larger, with a maximum difference of 32 mm, seen in head longitudinal (x) translation. For the y displacement, the difference was 9 mm (5 %) in the first phase and 9 mm (4%) in the second phase. In the first lane change sequence (approximately 0-1 s), the two models behaved similarly, with the largest difference in x translation (19 mm, 33%) and y rotation (7°, 72%), while in the second phase the two models start to deviate slightly more with the largest difference in x translation (32 mm, 68 %) and y rotation (10°, 60 %).


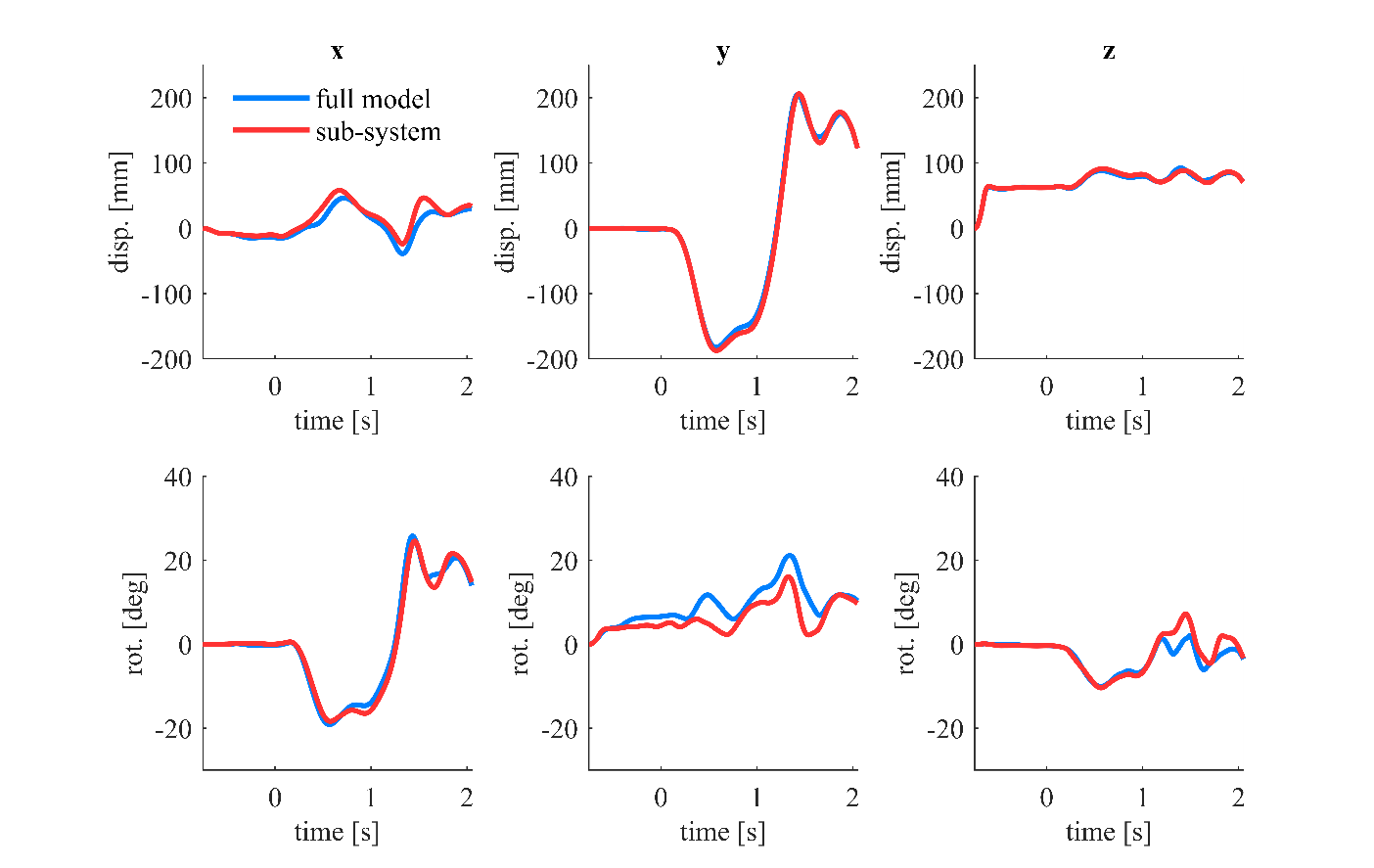


Figure S2. Comparison of head kinematics for full model (blue) and sub-system (red) in lane change simulations.

For the extracted sub-system, the dominating displacement for both braking (x direction) and lane chance (y direction) was matched with an excellent result, Figure S3. For braking, the rotation around x, with a rotation magnitude below 1°, Figure S1, was matched poorly, while the other directions were matched with a fair, good or excellent result. For lane change, the x translation and rotation around y and z was matched fairly, while the other directions were matched with an excellent result.


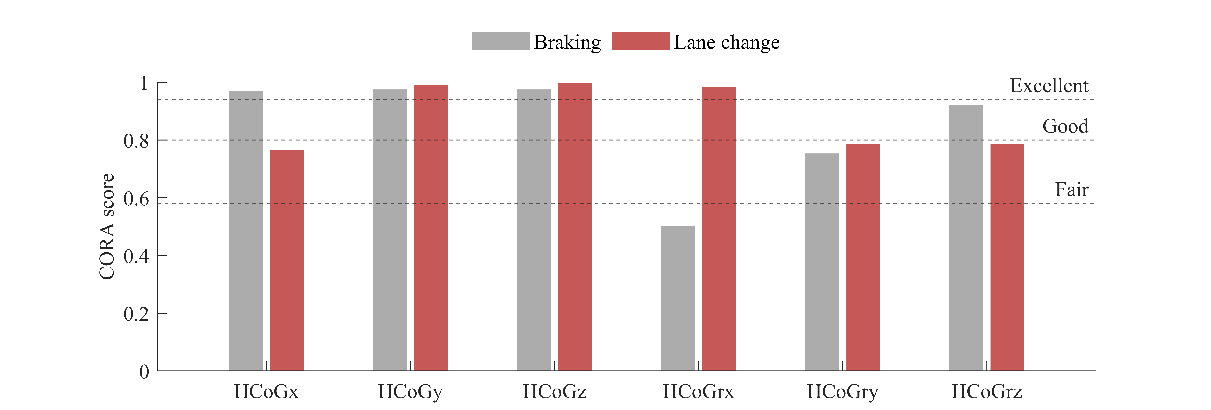


Figure S3. CORA scores for head kinematics comparing the sub-system signals to the full model signals for braking (gray) and lane change (red). The dotted lines indicate limits for CORA rating.

## Gain tuning

| 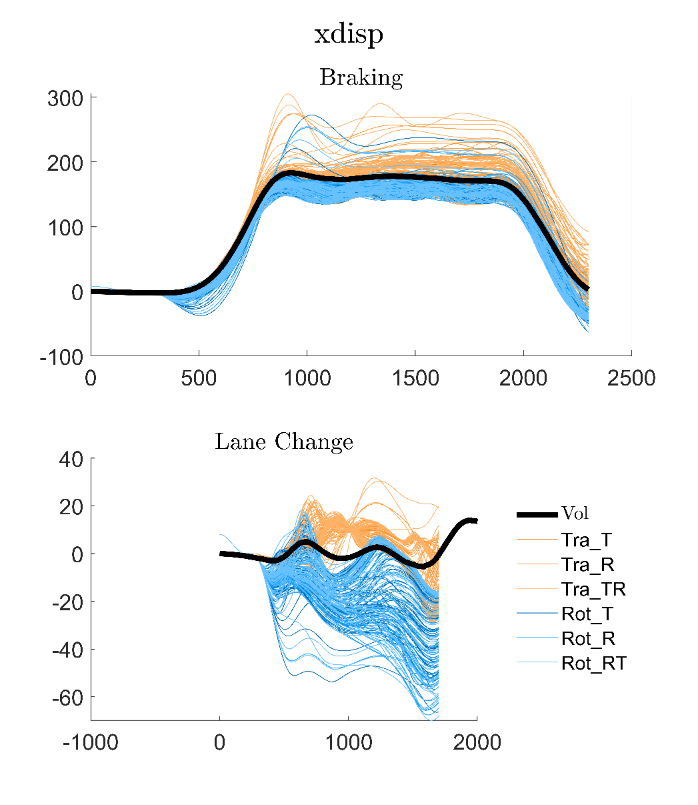 | 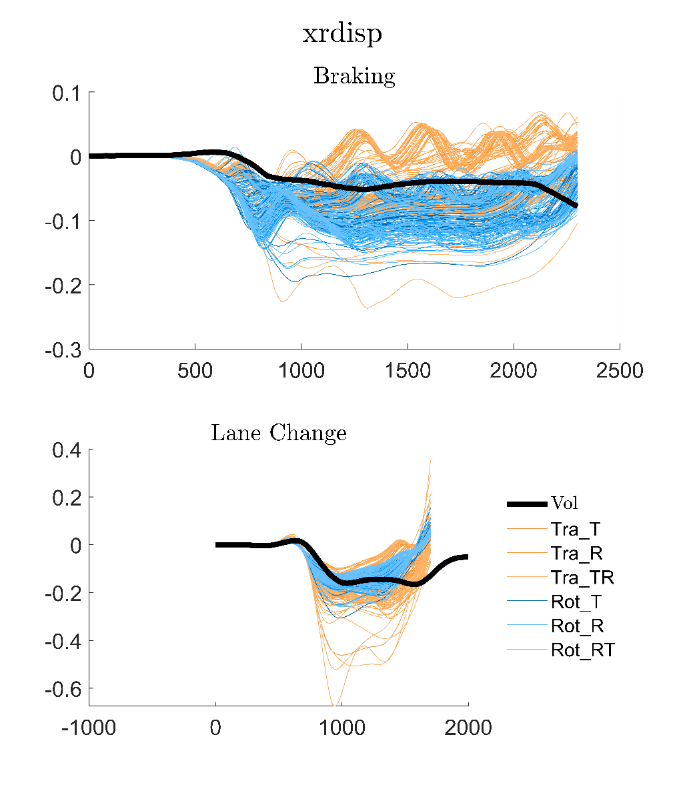 |
| --- | --- |
| 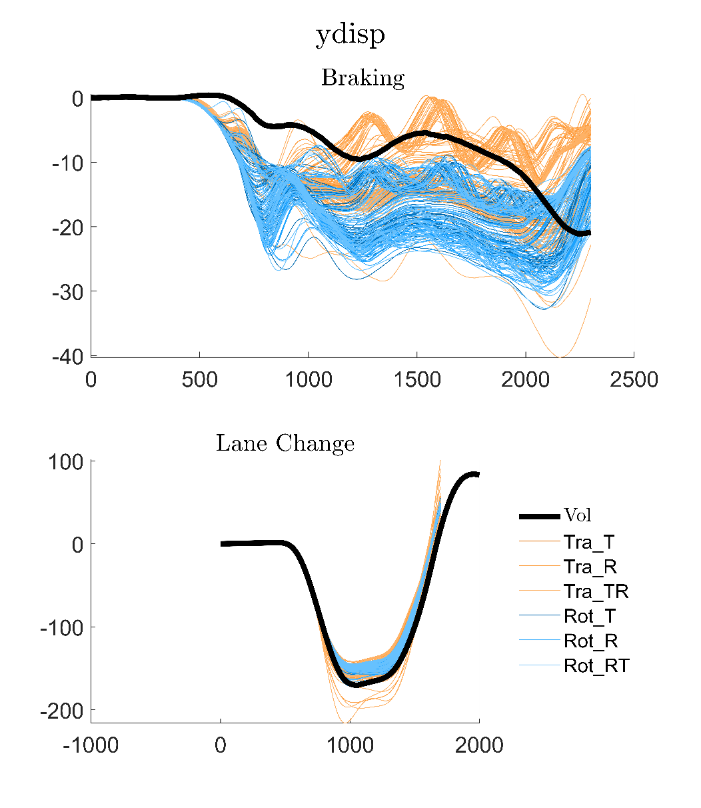 | 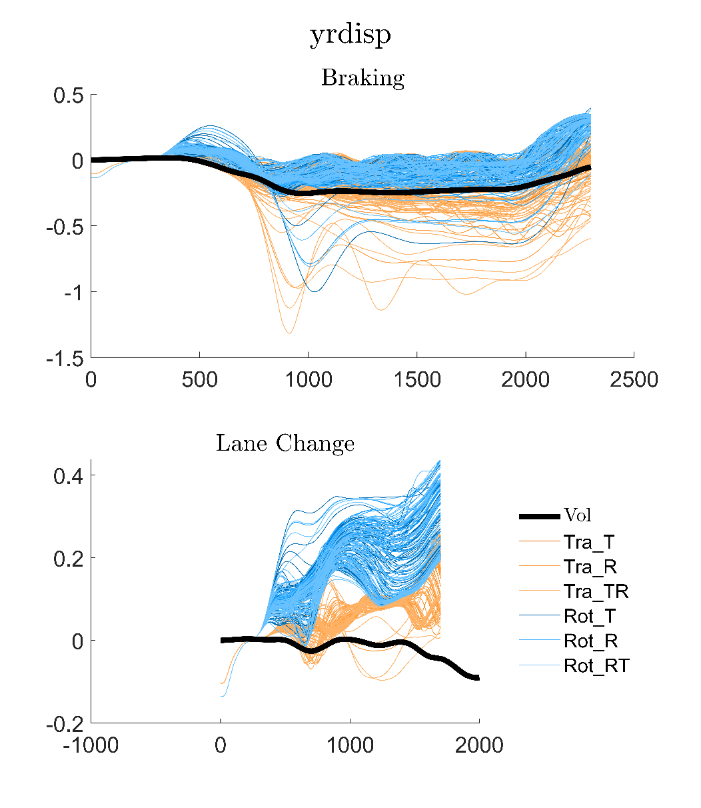 |
| 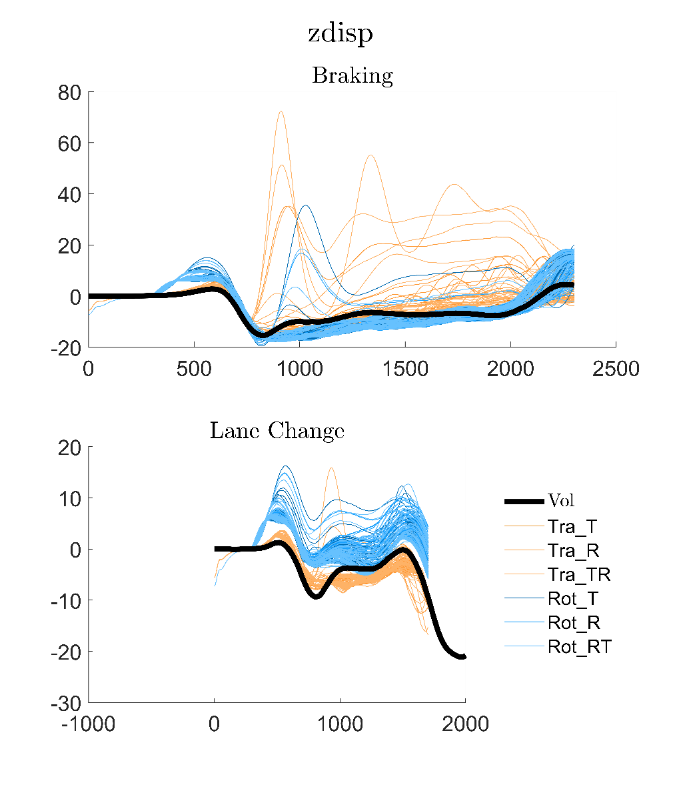 | 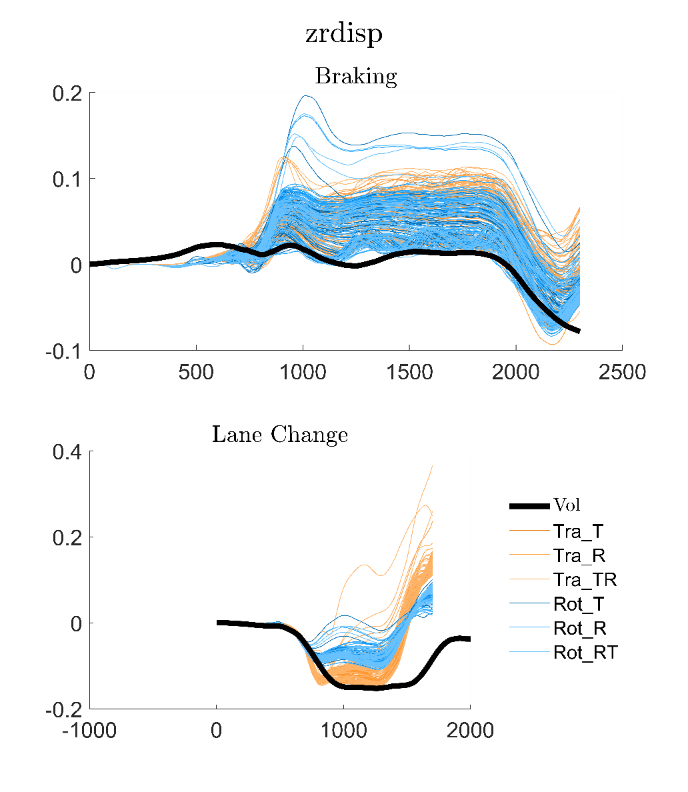 |

Figure S4. Head translations (left) and rotations (right) of all simulations in gain tuning (thin colored lines, orange for translational models and blue for rotational controller models) and volunteers (black thick line).

##
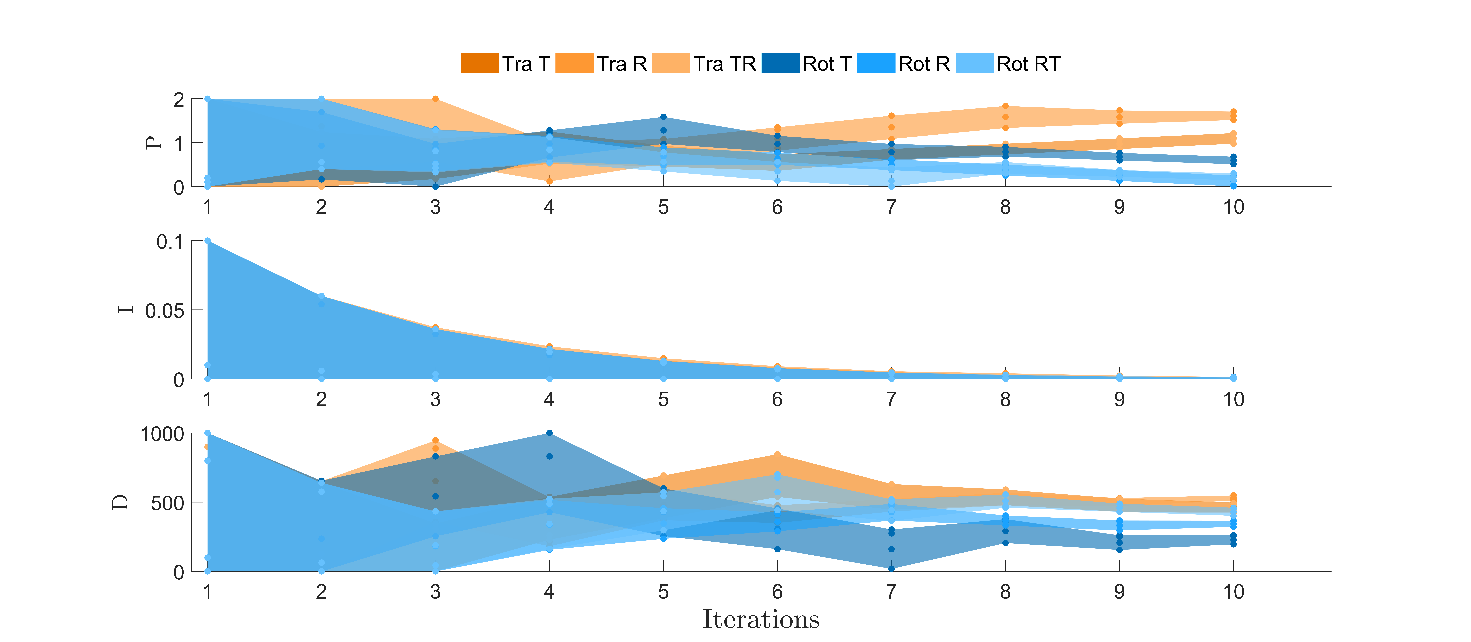


Figure S5. Convergence plots for K_p_, K_i_ and K_d_. Highlighted area shows upper/lower bounds for the optimizer, filled circles show evaluation points per iteration. Orange shows translational models and blue the rotational controller models.
